# An atypical and functionally diverse family of Kunitz-type cysteine/serine proteinase inhibitors secreted by the helminth parasite *Fasciola hepatica*

**DOI:** 10.1038/s41598-020-77687-7

**Published:** 2020-11-26

**Authors:** David Smith, Krystyna Cwiklinski, Heather Jewhurst, Irina G. Tikhonova, John P. Dalton

**Affiliations:** 1 School of Biological Sciences, Queen’s University Belfast, Belfast, BT9 7BL Northern Ireland, UK; 2 Centre of One Health and Ryan Institute, School of Natural Sciences, National University of Ireland Galway, Galway, Ireland; 3 School of Pharmacy, Medical Biology Centre, Queen’s University Belfast, Belfast, BT9 7BL Northern Ireland, UK; 4 Present Address: Moredun Research Institute, Pentland Science Park, Penicuik, Midlothian Scotland, UK

**Keywords:** Biochemistry, Molecular biology, Diseases

## Abstract

*Fasciola hepatica* is a global parasite of humans and their livestock. Regulation of parasite-secreted cathepsin L-like cysteine proteases associated with virulence is important to fine-tune parasite-host interaction. We uncovered a family of seven Kunitz-type (FhKT) inhibitors dispersed into five phylogenetic groups. The most highly expressed FhKT genes (group FhKT1) are secreted by the newly excysted juveniles (NEJs), the stage responsible for host infection. The FhKT1 inhibitors do not inhibit serine proteases but are potent inhibitors of parasite cathepsins L and host lysosomal cathepsin L, S and K cysteine proteases (inhibition constants < 10 nM). Their unusual inhibitory properties are due to (a) Leu^15^ in the reactive site loop P1 position that sits at the water-exposed interface of the S1 and S1′ subsites of the cathepsin protease, and (b) Arg^19^ which forms cation-π interactions with Trp^291^ of the S1′ subsite and electrostatic interactions with Asp^125^ of the S2′ subsite. FhKT1.3 is exceptional, however, as it also inhibits the serine protease trypsin due to replacement of the P1 Leu^15^ in the reactive loop with Arg^15^. The atypical Kunitz-type inhibitor family likely regulate parasite cathepsin L proteases and/or impairs host immune cell activation by blocking lysosomal cathepsin proteases involved in antigen processing and presentation.

## Introduction

*Fasciola hepatica,* the causal agent of fasciolosis, has the greatest geographical distribution and exhibits one of the broadest mammalian host ranges of all helminth (worm) parasites^1^. As a result, this disease afflicts millions of humans and their livestock on every inhabited continent^2^. Part of the parasite’s success can be attributed to its ability to secrete a rich source of proteins that aid its invasion of the host, penetration and feeding of tissues, as well as counteracting and downplaying host immune responses^2–4^. The best characterised are the abundantly expressed and secreted cathepsins L and B proteases that have 23 and 11 members, respectively, with overlapping and distinct substrate and macromolecular specificities^5^. Together, these secreted cysteine proteases create a formidable digestive cocktail that allows the parasite to efficiently and rapidly tunnel through host intestinal and liver tissues during its migration to the bile ducts^5,6^.

Regulation of the secretory protease activity is essential for parasites and is primarily achieved by the co-secretion of protease inhibitors^7–9^. In our search for protease inhibitors in *F. hepatica* secretions we discovered a small molecular-sized protein (6 kDa) that possessed all the structural features of Kunitz-type (FhKT1) inhibitors required to inhibit serine proteases^10^. To our surprise, however, the FhKT1 was unusual amongst KT inhibitors, as it exhibited no inhibitory activity against a wide range of standard serine proteases, including trypsin, chymotrypsin, elastase and various blood-clotting factors. Unexpectedly, FhKT1 possessed specificity and potent activity (Ki < 0.1 nM) against *F. hepatica* cathepsin L cysteine proteases as well as cathepsins L and cathepsins K of mammals and thus represented a specific evolutionary adaptation in their function never described previously for a KT inhibitor/I2 family inhibitor.

By interrogating the *F. hepatica* genome, transcriptome and proteome^3,4,11^ we have uncovered a wider family of KT inhibitor genes containing five groupings or clades (termed FhKT1–FhKT5) that are differentially expressed according to the parasite’s development in the host. The FhKT1 group, containing three members, are the most highly expressed, particularly within the early infective stages of the parasite and the only members secreted by the parasite, acting at the host-parasite interface. The FhKT1 clade are associated with the secretory cells of the parasite gut reproductive structures of mature adult parasites. Functional expression of recombinant FhKT1 together with mutagenesis studies show that a leucine present at position 15 (P1^15^) within the RSL is critical to defining the exclusive cysteine protease specificity of FhKT1.1, while arginine at this position confers FhKT1.3 with the ability to inhibit cysteine proteases and the serine protease trypsin. Using structural modelling we also determined that position 19 (P4′) is important in stabilising the RSL within the cysteine protease active site. Our studies provide important insights on how helminth parasites have a capacity to create novel molecules with unique and varied biological activities important in host-parasite interaction by the process of gene duplication and positive selection.

## Results

### *F. hepatica* Kunitz-type inhibitors are encoded by a multigene family

**Figure 1:**
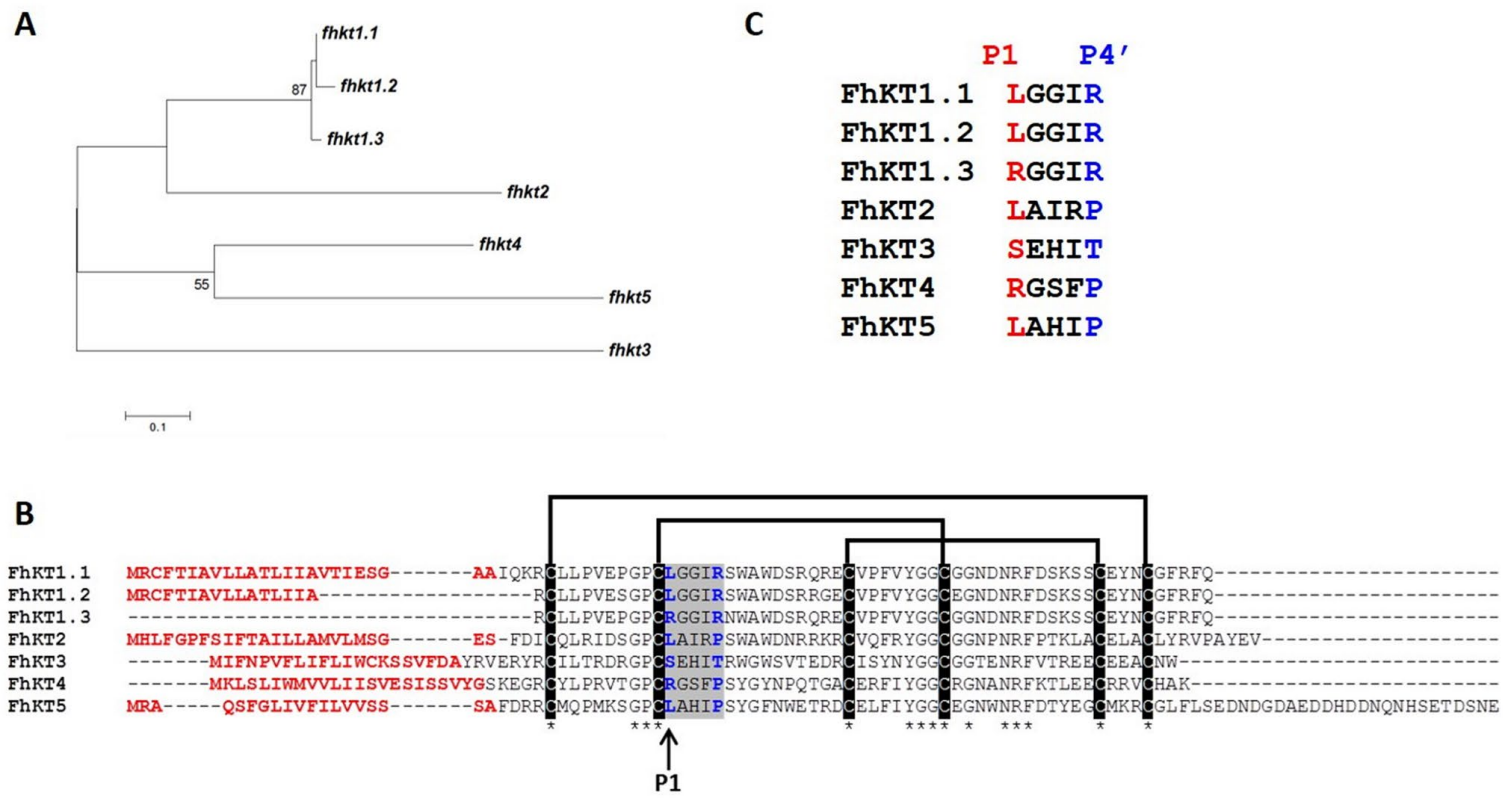
A family of Kunitz-type inhibitors is present in the *F. hepatica* genome. (**A**) Maximum-likelihood phylogenetic tree based on sequences encoding the kunitz domain in *F. hepatica* genome. Bootstrap values > 50% from 1000 replicate iterations are shown. (**B**) Amino acid sequence alignment of the translated amino acid sequence of each *fhkt* gene. The P1, residue 15, within the reactive site loop is indicated with an arrow. Secretory signal peptides are shown in red and lines show the three conserved disulphide bonds that occur between the six conserved cysteine residues, highlighted in black, specifically between Cys^1^ and Cys^6^, Cys^2^ and Cys^4^, and Cys^3^ and Cys^5^. The Kunitz reactive loop region is shaded in grey and P1 and P4′ residues predicted to interact with residues at the S2 and S2′ sites of cathepsin L-like cysteine proteases are highlighted in blue. (**C**) Amino acid sequence alignment of the reactive loop region of the seven members of the Kunitz family. The P1 and P4′ residues are highlighted red and blue, respectively.

By interrogating the *F. hepatica* genome we discovered a family of KT inhibitor genes consisting of seven members. Phylogenetic analysis showed that three of these genes clustered closely together, here termed the *fhkt1* group (*fhkt1.1, fhkt1.2 and fhkt1.3*), with the remaining individual genes forming distinct branches, termed *fhkt2*, *fhkt3*, *fhkt4* and *fhkt5* (Fig. 1A). Analysis of the genomic organisation of these genes shows that *fhkt1.1. fhkt1.2, fhkt1.3* and *fhkt5* are present on the same draft *F. hepatica* genome scaffold, with *fhkt2, fhkt3* and *fhkt4* present on separate draft genome scaffolds. With the exception of *fhkt1.3*, all genes are comprised of two exons, representing the signal peptide and KT domain, respectively, separated by an intron of varying length (ranging from 500 bp to > 10 kbp; Supplementary Fig. S1). The *fhkt1.3* sequence lacks the first signal peptide-encoding exon. Sequence analysis of 20 cDNAs amplified from adult *F. hepatica* cDNA using a forward primer encoding a consensus FhKT1 signal peptide sequence and a reverse primer corresponding to the conserved KT domain did not find any *fhkt1.3* gene. This supports the idea that this gene lacks a sequence associated with the first exon and, therefore, is deprived of a signal peptide.

Sequence alignments showed that six conserved cysteine residues, which form three characteristic disulphide bridges, are preserved in all seven FhKT proteins (Fig. 1B). Low primary sequence identity was observed between the sequences (21.1%; 12 residues) but of particular note was the significant variability within the P1–P4′ reactive loop region that is responsible for inhibitory function by KTs (Fig. 1C) that indicates important functional diversity and adaptation within the family of inhibitors. Strikingly, FhKT1.1, FhKT1.2, FhKT2 and FhKT5 possess a P1 leucine residue that we have shown is critical for the unique inhibition of cysteine proteases^10^, while FhKT1.3 and FhKT4 possess a P1 arginine residue, which is more typical of classical KT inhibitors that inhibit trypsin-like and chymotrypsin-like serine proteases^12–14^.

Interrogation of the genomes available for related important human and animal parasites revealed a surprisingly high number of diverse KTs that separate into seven groups (although smaller clusters exist within these groupings) based on maximum likelihood phylogenetic analysis (Supplementary Fig. S2, Supplementary Table S1). The functional diversity of the trematode KT inhibitors is further highlighted by the range of P1–P4′ reactive loop region sequences (Supplementary Table S1). In particular, this analysis demonstrated that the majority of sequences in Group A and C possess a P1 leucine residue, while Group B is dominated by sequences with a P1 arginine residue.

Surprisingly, despite the presence of a large number of expanded families of KTs from a diverse range of trematodes in Group D, F and G, these did not contain any sequences from *F. hepatica* (Supplementary Fig. S2). The *fhkt1* (*fhkt1.1, fhkt1.2 and fhkt1.3*) and *fhkt2* sequences were found within Group A with KTs from *Fasciola gigantica* and *Echinostoma caproni* but there were no comparable sequences found within the bile dwelling flukes, *Clornorchis sinensis* and *Opisthorchis viverrini*, or the blood flukes, *Schistosoma haematobium*, *Schistosoma japonicum* and *Schistosoma mansoni*. The *fhkt3* gene clusters within Group B with sequences from *E. caproni, C. sinensis* and *Schistosoma* species, while *fhkt4* and *fhkt5* genes are found in Group E with sequences from *E. caproni, C. sinensis* and *O. viverrini*.

### The *F. hepatica* KT inhibitor family members are under strict control of temporal expression and secretion

**Figure 2:**
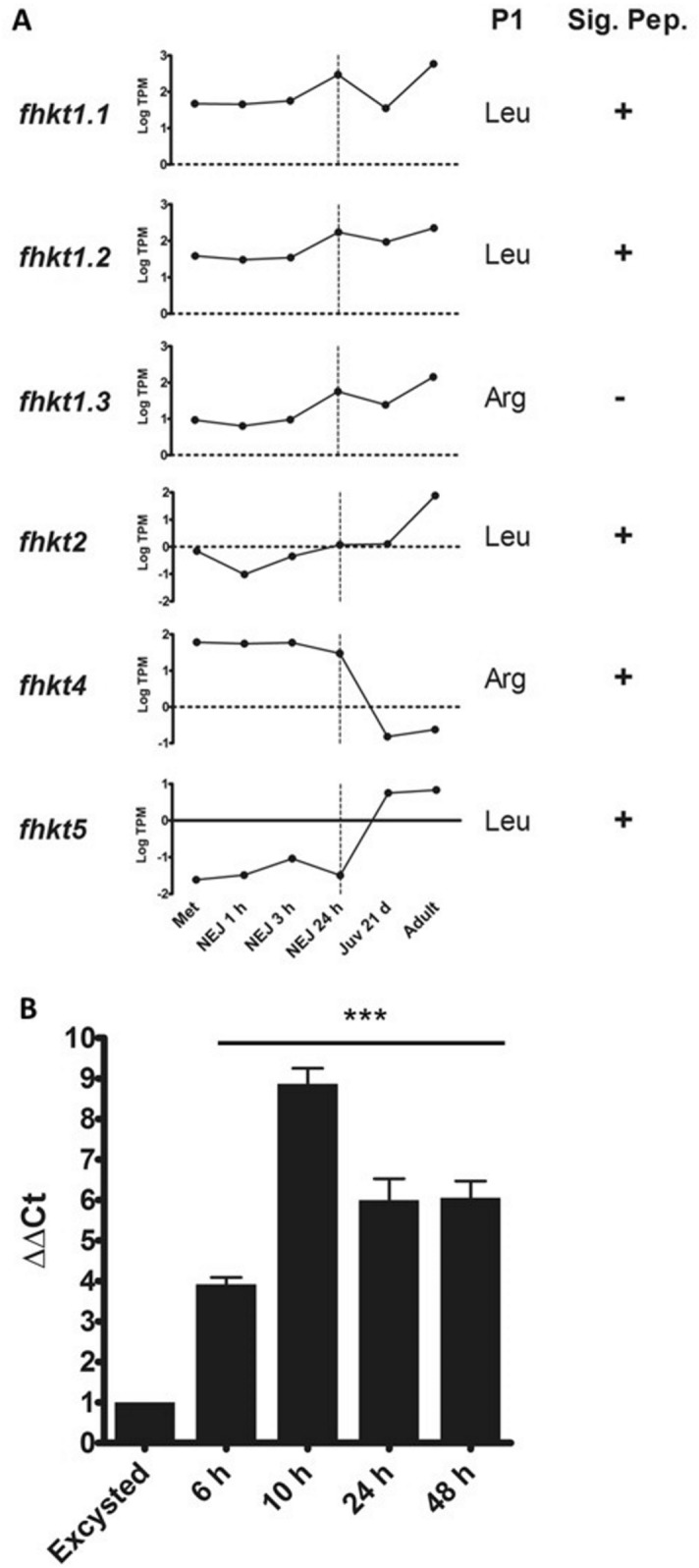
Temporal regulation of *F. hepatica* KT gene expression during the mammalian host-associated parasite stages. (**A**) Graphical representation using Graphpad software (v6 for Windows) of relative gene expression for each member of the *F. hepatica* KT family represented by log transcripts per million (log TPM) at various stages of the parasite’s lifecycle including the infective metacercariae (met), newly excysted juveniles (NEJ) at 1 h, 3 h and 24 h post-excystment, liver stage juvenile parasites 21-days post infection (Juv 21 d) and bile ducts stage mature adult parasites (adult). The encoded P1 residue and the absence or presence of a signal peptide (Sig. Pep) is shown alongside each graph. (**B**) Relative fold expression of the genes representing the *fhkt1* group during the first 48 h post-excystment by NEJ (normalised to expression at NEJ excystment and relative to a GAPDH reference, with SEM). Statistical analysis was carried out using One Way ANOVA with Tukey’s post hoc test, with all samples showing statistical differences in fold expression compared with the levels at excystment (*p* < 0.001: ***), visualised using GraphPad Software (v6 for Windows).

Of the seven *F. hepatica* KT genes, the *fhkt1* group (*fhkt1.1, fhkt1.2 and fhkt1.3*) were found to be the most highly transcribed KT inhibitors in *F. hepatica* (Fig. 2A). Moreover, all three members exhibited a similar pattern of temporal transcription through the different development stages within the host and it was noteworthy that all were significantly transcribed at higher levels at 24 h post-excystment and also within the mature adult parasites, relative to the metacercariae (Fig. 2A; Supplementary Fig. S3). Quantitative gene expression analysis (qPCR), performed on the infectious encysted metacercariae and NEJs over a time-course of 48 h post-excystment, revealed a marked rise in *fhkt1* transcription, particularly at ~ 10 h post-excystment, a time when the parasite is traversing the host gut wall (Fig. 2B).

The *fhkt4* gene also exhibited a high level of transcription within the metacercariae and NEJ 1 h, 3 h and 24 h stages but in contrast to the *fhkt1* group, this gene is not expressed by the 21-day old immature parasites that migrate within the liver tissue and mature adult parasites that reside in the bile ducts (Fig. 2A). By contrast, transcription of *fhkt2* and *fhkt5* are not detected in the infectious NEJ stages but are required by the later stage parasites; *fhkt2* is up-regulated in mature adult flukes while *fhkt5* appears earlier in 21-day old as well as mature adult parasites, though at lower levels of transcription (Fig. 2A). Statistically relevant levels of transcription of *fhkt3* were not detected at any stage suggesting that *fhkt3* is either expressed in lifecycle stages not associated with the mammalian host (e.g. during invasion and migration in the intermediate snail host) or, perhaps less likely, is a pseudogene.

Analysis of the proteomic profiles of the secretomes of various stages of the parasite previously reported by us^4,11^ revealed that the FhKT1 group, FhKT1.1, FhKT1.2 and FhKT1.3, are the only members of the family that are secreted extra-corporeally by the parasite (Supplementary Table S2). Consistent with the higher levels of transcription within the 24 h NEJ and the adult parasites, these inhibitors were detected in the secretomes of the NEJ parasites (1 h, 3 h and 24 h post-excystment) and mature adult parasites. Furthermore, FhKT1.1 and FhKT1.2 were also detected amongst the cargo of proteins contained within the extracellular vesicles (EVs) prepared from the excreted secreted (ES) products of adult flukes (Supplementary Table S2, and^11^). Although FhKT1.3 lacks a signal peptide it was also found in the parasite secretions, which indicates that its delivery into the host is mediated by a non-classical pathway as observed for other *F. hepatica* proteins such as glutathione-S transferase^3,4,6^.

### Tissue-specific expression of KT inhibitors in juvenile and adult parasites

**Figure 3:**
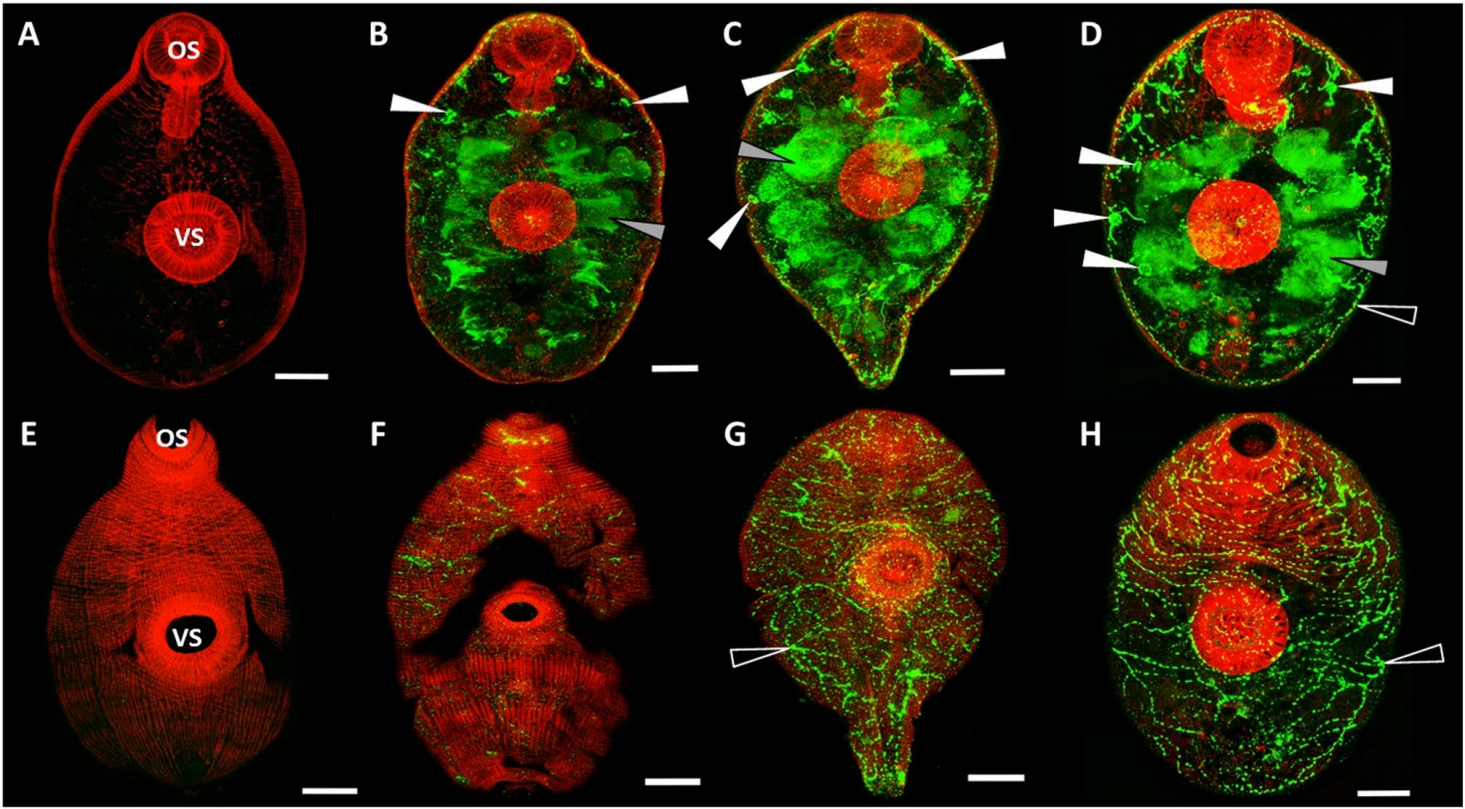
Immunolocalization of *F. hepatica* KT in infective NEJs in the first 48 h of infection. (**A**–**D**) show a plane within the interior of the NEJ, whereas (**E**–**H**) show a plane at the surface of the same individual NEJs. Following excystment, NEJs were maintained in culture and sample parasites taken at 3 h post-excystment (**B**,**F**), 24 h post-excystment (**C**,**G**) and 48 h post-excystment (**D**,**H**). Parasites were probed with rabbit pre-immune serum (**A**,**E**) or anti-FhKT1 antibodies followed by FITC-labelled secondary antibodies. The presence of FhKT within *F. hepatica*-specific structures are indicated by arrows; parenchymal cell bodies, white arrows; NEJ digestive tract, grey arrows; network of channels below surface tegument, black arrows. All specimens were counter-stained with phalloidin-tetramethylrhodamine isothiocyanate (TRITC) to stain muscle tissue (red fluorescence) and provide structure. *OS* oral sucker, *VS* ventral sucker. Scale bars 20 μM.

FhKT1 was immunolocalised to the NEJ parenchymal cell bodies and within the large bifurcated gut (Fig. 3B–D) which also expresses the major cysteine protease *F. hepatica* cathepsin L3 (FhCL3) and cathepsins B (FhCB). By altering the plane of visualisation to view the surface of the NEJ parasites, we observed a complex network of thin channels never described before on the underside of the NEJ surface tegument that connected the parenchyma cells. The intensity of anti-FhKT1 labelling within these intracellular channels was particularly abundant in the 24 h and 48 h NEJs (Fig. 3F-H, black arrowheads). The channels are not associated with the musculature nor do they have any obvious connection with the oral or ventral sucker; rather, the labelling suggests that they create a transport system between the parenchymal cell bodies. As a negative control, NEJ samples were also probed with pre-immune sera (Fig. 3A,E).

FhKT1 was also highly expressed in the adult parasite’s reproductive organs, particularly the vitelline glands, which are important for egg development, and in eggs within the ovaries (Supplementary Fig. S4). Antibody binding was also observed within the parenchyma and gut wall (Supplementary Fig. S5).

### Inhibition profile of the FhKT1 members and importance of P1^15^ residue in determining specificity

**Figure 4:**
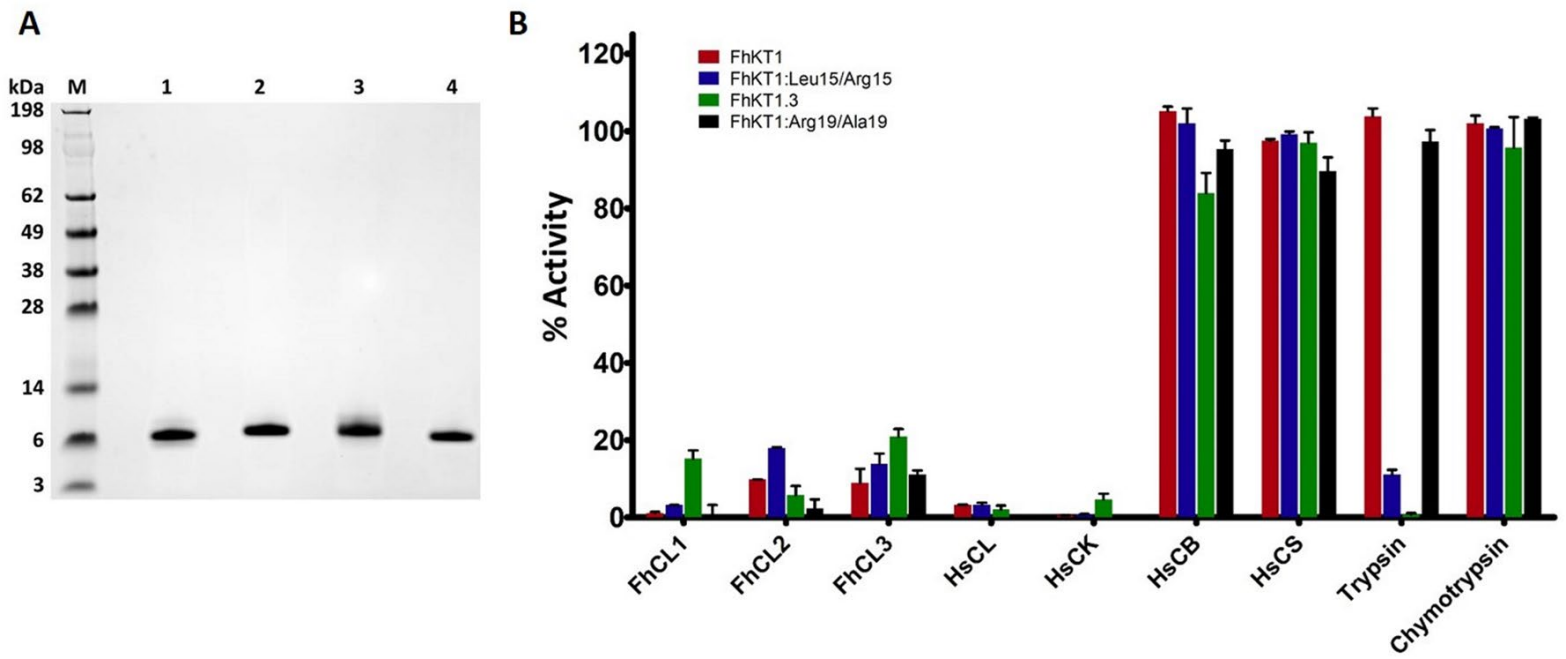
Purification of recombinant Kunitz-type inhibitors and their inhibition profiles against cysteine and serine proteases. (**A**) LDS-PAGE analysis of the recombinantly yeast-expressed *F. hepatica* KT inhibitors, rFhKT1.1 (lane 1); rFhKT1.1Leu^15^/Arg^15^ (lane 2); rFhKT1.3 (lane 3) and, rFhKT1Arg^19^/Ala^19^ (lane 4). M, molecular size markers. (**B**) Inhibitory activity (2 μM ) of rFhKT1.1 (red bars), rFhKT1Leu^15^/Arg^15^ (blue bars); rFhKT1.3 (green bars) and rFhKT1Arg^19^/Ala^19^ (black bars) against a range of cysteine proteases including *F. hepatica* cathepsin L1 (FhCL1), *F. hepatica* cathepsin L2 (FhCL2), *F. hepatica* cathepsin L3 (FhCL3), human cathepsin L (HsCL), human cathepsin K (HsCK), human cathepsin B (HsCB), human cathepsin S (HsCS) and the serine proteases trypsin and chymotrypsin. Inhibition is presented relative to the activity of each enzyme in the absence of inhibitors ± SD, visualised using GraphPad Software (v6 for Windows).

Recombinant FhKT1.1 (rFhKT1.1) was produced as a functionally active recombinant protein and isolated to homogeneity by NTA-agarose affinity chromatography (Fig. 4A; Supplementary Fig. S7). rFhKT1.1 was previously shown to be a potent inhibitor of two major cysteine proteases secreted by adult *F. hepatica*, FhCL1 and FhCL2, although the inhibition constant, K_i_, is about 25-fold more for the latter enzyme (Table 1^10^). In the present study, we found that rFhKT1.1 is also a potent inhibitor of the NEJ-specific cathepsin protease, FhCL3, an enzyme that has a unique activity and can digest interstitial matrix proteins such as collagen; a reduction of activity of 93.1% (± 3.66) was observed at 2 μM inhibitor and we determined a K_i_ value of 1.8 nM (± 0.6) (Fig. 4B, Table 1). rFhKT1.1 is also a potent inhibitor of the human cathepsin L-like cysteine proteases human cathepsin L and K, with similar potencies to that observed for the parasite proteases (Table 1). By contrast, rFhKT1.1 exhibits no activity against a range of serine proteases including trypsin and chymotrypsin (Fig. 4B, Table 1) and kallikrein, thrombin, plasmin and elastase (data not shown).

**Table 1:** Inhibition constants (K_i_) for the FhKT recombinant protein against cysteine and serine proteases inhibited.

Enzyme	Inhibition constant (K_i_), in nM			
	rFhKT1^a^	rFhKT1Leu^15^/Arg^15^	rFhKT1.3	rFhKT1Arg^19^/Ala^19^
**Cysteine proteases**				
*F. hepatica* Cathepsin L1	0.4 (± 0.1)^a^	0.7 (± 0.04)^a^	0.6 (± 0.2)	4.2 (± 0.4)
*F. hepatica* Cathepsin L2	10 (± 0.3)^a^	27 (± 1.5)^a^	10.6 (± 0.3)	35 (± 3.9)
*F. hepatica* Cathepsin L3	1.8 (± 0.6)	3.6 (± 0.3)	3.2 (± 0.2)	32.8 (± 7)
Human Cathepsin L	1.6 (± 0.1)^a^	3 (± 0.1)^a^	2.6 (± 0.5)	0.3 (± 0.004)
Human Cathepsin K	5 (± 0.3)^a^	5 (± 0.3)^a^	5.5 (± 0.3)	18.5 (± 2.9)
**Serine protease**				
Bovine Trypsin	N.I	1.5 (± 0.7)^a^	1.8 (± 0.2)	N.I

*N.I* not inhibited.

^a^As reported by Smith et al.^10^.

The residue at the P1 site (position 15) is centrally poised within the reactive loop of KT inhibitors and is critical for binding to and inhibiting the target protease. Sequence alignment analysis shows that the P1 position in two members of the *F. hepatica* FhKT1 group, FhKT1.1 and FhKT1.2, is occupied with a hydrophobic leucine residue (see Fig. 1C). By comparison, FhKT1.3 possesses a positively charged arginine in this position. We therefore produced a recombinant form of this inhibitor, rFhKT1.3, and demonstrated that it was also a potent inhibitor of the *F. hepatica* cathepsin L proteases rFhCL1, rFhCL2, rFhCL3, with K_i_ of 0.6, 10.6 and 3.2 nM, respectively. Likewise, rFhKT1.3 was also a potent inhibitor of human cathepsins K and L with K_i_ of 2.6 and 5.5 nM, respectively. However, unlike FhKT1.1 and FhKT1.2, rFhKT1.3 showed potent inhibitory activity against the serine protease trypsin with a reduction in activity of 99.23% (± 0.36) at 2 μM and a K_i_ of 1.8 nM (± 0.2). rFhKT1.3 did not inhibit other serine proteases tested, such as chymotrypsin (Fig. 4B, Table 1).

This inhibitory profile of FhKT1.3 (i.e. potent activity against both cysteine and serine proteases) is comparable to that exhibited by a variant of FhKT1.1 whereby the PI Leu^15^ was purposely substituted with an P1 Arg^15^ to produce the recombinant rFhKT1.1Leu^15^/Arg^15^ (see Table 1;^10^). This data emphasises the importance of the amino acid residue at the P1 site of the reactive loop in inhibition specificity.

### Arg^19^ in the C-terminus of the reactive loop of FhKT1 is important for binding to cysteine proteases

**Figure 5:**
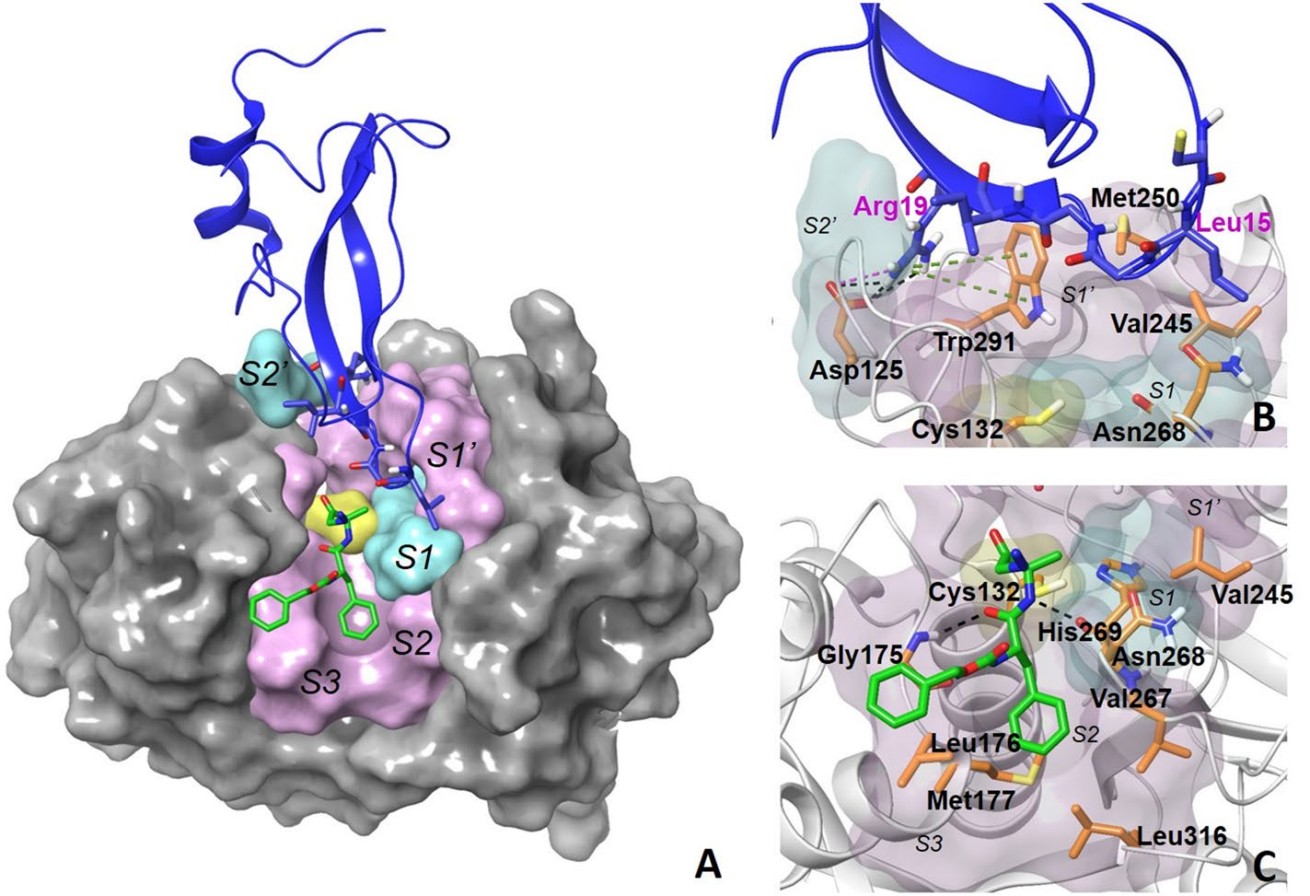
3-D model of FhCL1 interactions with FhKT1.1 and Z-Phe-Ala-CHN_2_. (**A**) The overall view of the 3-D model of the FhCL1 tertiary complex in surface representation. Regions forming the S1, S2, S1′ and S2′ active site subsites of FhCL1 are shown in alternating pink and cyan surface. The reactive Cys residue within the S1 pocket of the cysteine protease is shown in yellow. FhKT1 is shown by the dark blue cartoon with the reactive site loop (Leu^15^-Arg^19^) in a stick-like representation, whereas Z-Phe-Ala-CHN_2_ is shown by the green stick representation. (**B**) FhKT1.1 reactive site loop residues Leu^15^-Gly^16^-Gly^17^ are shown in stick format (dark blue) within the active site of the *F. hepatica* cathepsin L1. Leu^15^ sits near Val^245^ of S1′ and Asn^268^ of S1. Arg^19^ forms hydrogen bonds and salt bridge interactions with Asp^125^ and cation-π interactions with Trp^291^, shown in by the dotted lines in black, pink and green, respectively. (**C**) The cysteine protease inhibitor Z-Phe-Ala-CHN_2_ is shown in stick format (green) within the active site of the *F. hepatica* cathepsin L1. The inhibitor forms hydrogen bonds with the backbone of Asn^268^ and Gly^175^ from the S1 and S2 subsites, respectively, depicted by the black dotted lines. Graphical representations were created using Schrödinger software (LLC. Maestro, Version 2018-4; https://www.schrodinger.com/).

Homology models of FhKT1.1 docked to the crystal structure of FhCL1 (PDB code: 2O6X;^10^) (see Fig. 5A,B) were used to assess the shape and electrostatic interactions that take place between the two molecules. These predicted that an Arg residue situated at P4′ at the C-terminal end of the reactive loop (residue 19, see Fig. 1C) forms cation-π interactions with Trp^291^ of the S1′ subsite and electrostatic interactions with Asp^125^ of the S2′ subsite of the active site of the FhCL1 cysteine protease. To investigate the role of this residue in cysteine and serine protease binding of FhKT1 we produced a recombinant variant inhibitor whereby the positively charged arginine was replaced with a neutral alanine residue (rFhKT1.1Arg^19^/Ala^19^; Fig. 5A). The purified recombinant protein rFhKT1.1Arg^19^/Ala^19^ was shown to be a potent inhibitor of the *F. hepatica* cysteine proteases at 2 μM. However, K_i_ values of 4.2, 35 and 32.8 nM against rFhCL1, rFhCL2 and rFhCL3, respectively, demonstrated that this substitution reduced the potency of the inhibitor by 10-, 3.5- and 18-fold, respectively, compared to wild-type FhKT1 (Table 1). The K_i_ value of rFhKT1.1Arg^19^/Ala^19^ against human cathepsin K was also increased, 3.5-fold compared to wild-type FhKT1.1 but, surprisingly, the K_i_ for human cathepsin L was reduced fivefold and therefore binding was improved. Like wild-type FhKT1.1, rFhKT1.1Arg^19^/Ala^19^ showed no activity against bovine trypsin (Table 1) or other serine proteases examined (data not shown).

### FhKT1.1 and FhKT1.3 are potent inhibitors of native *F. hepatica* somatic and secreted cathepsin L cysteine proteases

**Figure 6:**
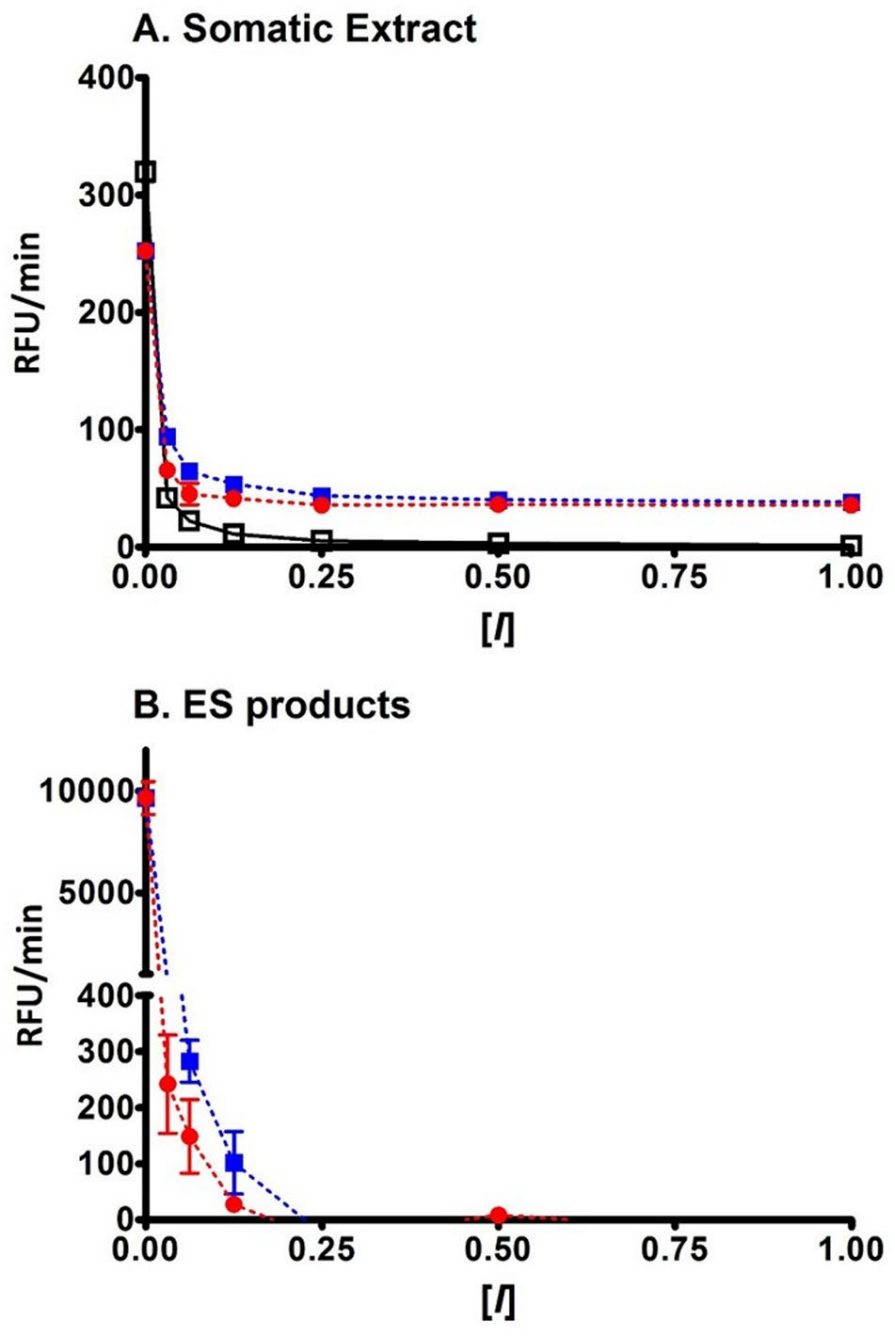
*F. hepatica* KT inhibitors inhibit all secreted cysteine protease activity, but not somatic extract activity. (**A**) Cysteine protease activity within somatic extracts of adult *F. hepatica* were measured using the fluorogenic peptide substrate Z-Phe-Arg-NHMec (relative fluorescent units, RFU/min) in the presence of FhKT1.1 (red line), FhKT1.3 (blue line) and the cysteine protease inhibitor E-64 (black line) at a range of concentrations [*I*] (1 μM, 500 nM, 250 nM, 125 nM, 62.5 nM, 31.25 nM and 15.625 nM). The *F. hepatica* KT inhibitors do not completely inhibit all cysteine protease within the somatic extract. (**B**) Cysteine protease activity within the ES proteins of adult *F. hepatica* measured in the presence of FhKT1.1 (red line) and FhKT1.3 (blue line) at a range of concentrations [*I*] (1 μM, 500 nM, 250 nM, 125 nM, 62.5 nM, 31.25 nM and 15.625 nM). Graphical representations produced using GraphPad Software (v6 for Windows).

Adult *F. hepatica* parasites express abundant cathepsin B and cathepsin L cysteine proteases and the major enzymes, FhCL1, FhCL2 and FhCL5, are also excreted/secreted into the culture medium in which the parasites are maintained. We examined whether recombinant FhKT1.1 and FhKT1.3 could inhibit these enzymes in somatic extract and ES products. Inhibition curves showed that cysteine protease activity in somatic extracts was inhibited by FhKT1.1 and FhKT1.3 but this was not absolute, even when the inhibitors where added to the extract at a 1 μM concentration (~ 20% activity remained, Fig. 6A). Total cysteine protease activity in the somatic extract, however, was completely inhibited by the broad-spectrum cysteine protease inhibitor E-64 (at concentrations above 250 nM; Fig. 6A). On the other hand, FhKT1.1 and FhKT1.3 completely inhibited cysteine protease activity within the ES products at concentrations of ~ 250 nM and above (Fig. 6B). We can explain this finding by our previous data showing that FhKT1.1 inhibits cathepsin L activity but not cathepsin B activity^10^; while cathepsin B cysteine proteases are present within the parasite somatic extracts of adult *F. hepatica,* they are not abundantly secreted by the parasite into culture medium^15–18^.

### Competition assays show that FhKT1.1 binding to cathepsin L is not blocked by small-molecule inhibitors of cysteine proteases that occupy S1, S2 and S3 subsites

**Figure 7:**
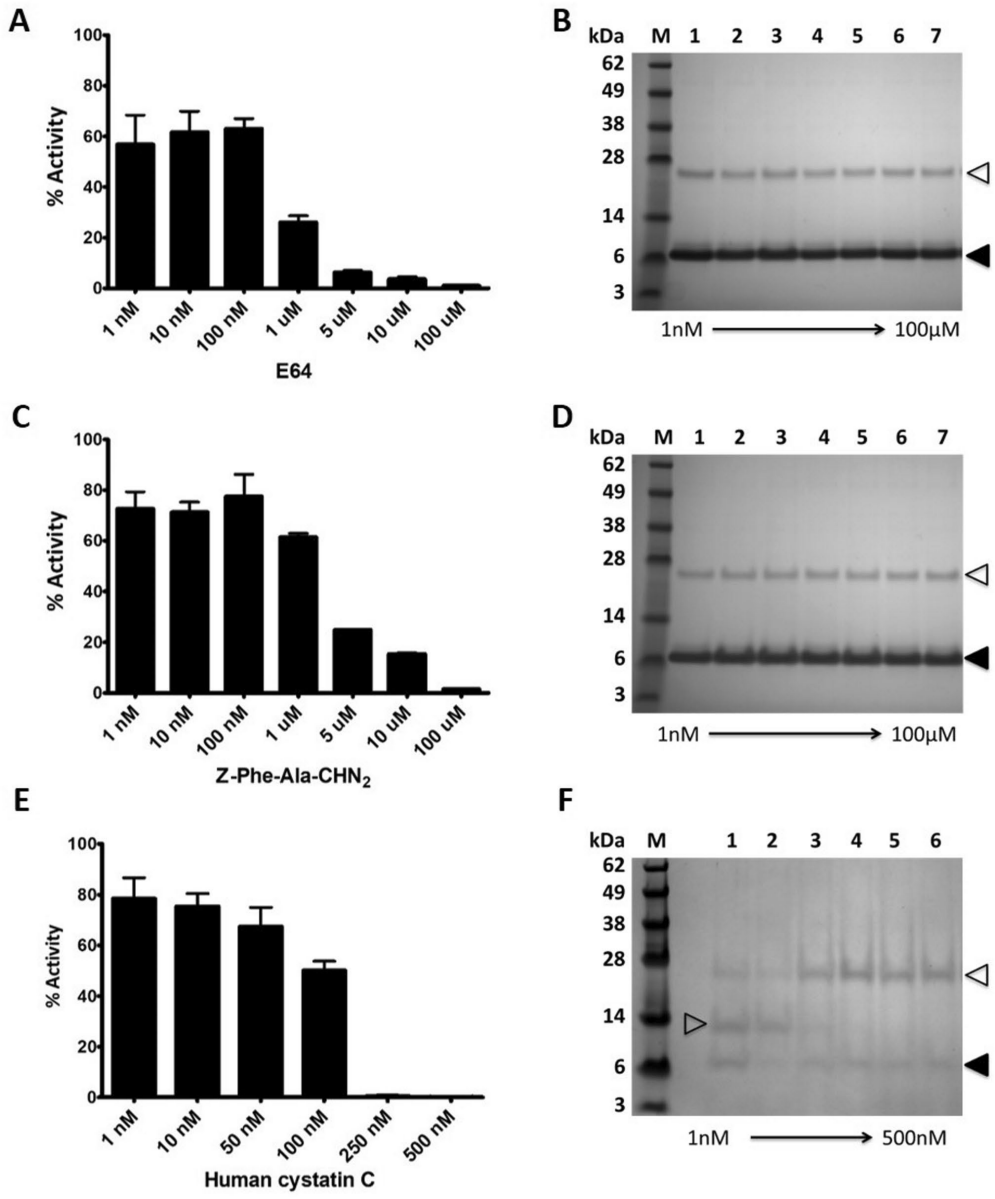
rFhKT1.1 binds to the active site groove but not within the active site pockets. Cysteine protease activity in adult *F. hepatica* excretory/secretory (ES) products measured in the presence of increasing concentrations of cysteine protease inhibitors E-64 (**A**), Z-Phe-Ala-CHN_2_ (**C**) and human cystatin C (**E**) (% activity, relative to the cysteine protease activity of ES containing no inhibitor ± SD). rFhKT1.1 (10 μM) was added to replicate reaction samples and then pull-down using NTA-beads (**B**,**D**,**F**). In the presence of the low molecular weight inhibitors, E-64 (**B**) and Z-Phe-Ala-CHN_2_ (**D**), rFhKT1.1 (black arrows, **B**,**D**) is not prevented from interacting with the cathepsin L cysteine proteases (white arrows, **B**,**D**). By contrast, interactions between rFhKT1.1 (black arrow, **F**) and cathepsin cysteine protease (white arrow, **F**) are blocked by the recombinant human cystatin C (grey arrow, **F**) observed by LDS–PAGE. Graphical representations produced using GraphPad Software (v6 for Windows).

To gain insight into the mechanism by which FhKT1.1 inhibits cysteine proteases we performed competition studies with small-molecule broad-spectrum inhibitors of cathepsin-like proteases, namely E-64 and Z-Phe-Ala-CHN_2_^19,20^ (Fig. 7; Supplementary Fig. S7). These assays involved first mixing the small compound at varying concentrations with adult *F. hepatica* ES products containing native cysteine proteases before adding the rFhKT1.1 inhibitor. The complex was subsequently pulled down using NTA-beads and then analysed by LDS-PAGE. Addition of E-64 or Z-Phe-Ala-CHN_2_ to the ES products_,_ even at excess concentrations of 100 μM, did not prevent the binding of rFhKT1.1 to the native secreted cysteine proteases (Fig. 7A–D). This data demonstrates that when small molecular-sized inhibitor compounds occupy the S1 and S2 subsite of the active site, FhKT1.1 can still bind to the active site groove of the cysteine proteases (Fig. 5A,C). By comparison, competition assays using recombinant human cystatin C, a cysteine protease inhibitor of 13.3 kDa, showed that this prevented the binding of FhKT1.1 to the active site groove (Fig. 7C). Similar observations were made with rFhKT1Arg^19^/Ala^19^ using Z-Phe-Ala-CHN_2_ (Supplementary Fig. S6).

## Discussion

From infection via the intestine as a newly excysted juvenile to establishment as a mature parasite in the bile ducts the helminth parasite *F. hepatica* embarks on a migratory path that requires the penetration and degradation of various tissues. Digestion of the various macromolecules encountered during this journey is accomplished by the controlled secretion of distinct members of a complex family of cathepsin-cysteine proteases. Control of these proteases to prevent excessive damage to parasite and host implies the involvement of inhibitors that regulate their activity and prevent excessive hydrolysis, which is often coined the protease/anti-protease balance^21,22^.

In this study, we found that *F. hepatica* expresses a family of Kunitz-type inhibitors consisting of seven members. Based on phylogenetic analysis, three of these genes, *fhkt1.1*, *1.2* and *1.3*, form a single gene cluster while the four remaining genes are distinct, namely *fhkt2*, *fhkt3*, *fhkt4* and *fhkt5*. Gene expression analysis using RNA-Seq data revealed that *fhkt1.1*, *1.2* and *1.3* are highly expressed at all intra-mammalian stages examined, from the NEJs that initiate infection by penetrating the gut wall to the bile duct-dwelling mature adult worms. qPCR showed that within 6–10 h after the NEJ parasites emerged from their cysts, expression of *fhkt1.1*, *1.2* and *1.3* are rapidly up-regulated. Furthermore, proteomic analysis of NEJ and adult worm secretions (ES products) and EVs detected peptides that match only proteins derived from the FhKT1.1, 1.2 and 1.3 gene products. Collectively, these observations suggest that the FhKT1 group of Kunitz-protease inhibitors are the most dominant KT inhibitors in the parasite stages examined and imply their importance in the interaction with the mammalian host, particularly during the early invasive process.

The *fhkt4* gene is expressed primarily in the metacercariae and in vitro-cultured NEJs but is markedly downregulated in parasites that have migrated and matured in the liver parenchyma (21 days after infection). The encoded FhKT4 protein differs to the FhKT1 group of kunitz-protease inhibitors as it has a P1 Arg and a P1′ Ala, which predicts that this inhibitor may inhibit trypsin (see below). As trypsin is a major digestive serine protease in the intestine, it could be harmful to the parasite as it excysts in the duodenum and begins migration across the intestinal wall. In *Fasciola gigantica*, the FhKT4 homolog, is abundantly transcribed by the cercarial stage within the snail^23^, indicating a possible role for the inhibition of trypsin-like proteases that are released by snails with cercariae, as shown in *Schistosoma mansoni*^24^. Therefore, FhKT4 may be specialised for anti-trypsin defence both within the intestine and in the snail intermediate.

Although, products of the *fhkt2* and *fhkt5* genes were not found in our proteomic analysis of parasite somatic and secreted proteins, transcriptomic data showed that their expression in the parasite life cycle is strictly regulated. They are both up-regulated in the migrating liver stage 21-day old parasites and in the bile duct-dwelling adult worms. Their expression, therefore, is associated with the tissue and blood-feeding life stages of *F. hepatica* and thus we propose functions in anti-coagulation, similar to that suggested for KT inhibitors of the trematodes *S. japonicum* and *S. mansoni*^25,26^. No transcript data was identified for the *fhkt3* gene which suggests it is either (a) expressed in life stages not associated with the mammalian host i.e. the intermediate snail host, or (b) is redundant and not expressed in *F. hepatica*.

Confocal immunolocalisation studies using antibodies prepared against recombinant FhKT1 showed that the FhKT1 group members were expressed in the gut lumen of the NEJ and within distinct parenchymal cell bodies. They are also observed in narrow channels that form a network throughout the parasite and penetrate the underside of the surface tegument (but do not appear to protrude into the tegument). Therefore, FhKT1 could be trafficked via these parenchymal cell bodies to different sites within the parasite, most predominantly to the digestive tract. From here FhKT1 proteins may be secreted by the parasite, explaining the presence of FhKT1.1, 1.2 and 1.3 peptides within NEJ ES products, and delivered into host cells and/or tissues. It is worth noting that because of the close identity in sequence/structure of the three FhKT1 members, as well as FhKT4, the polyclonal antibodies employed in our studies likely bind epitopes in all four inhibitors and thus the pattern of tissue localisation represents a composite of these proteins. Thus, different Kunitz-type inhibitors could be expressed in the cell bodies, parenchymal tissue and the digestive tract.

In adult parasites, FhKT1 group members are predominantly associated with structures of the female reproductive system including vitelline cells within the vitelline glands, S1 secretory cells of the Mehlis gland and within the vitelline of the eggs. While two of the major cathepsin L proteases expressed by adult *F. hepatica*, FhCL1 and FhCL2, are associated with the digestive tract and have not been localized in vitelline cells or parasite eggs^5,27^, another adult-associated cysteine protease, FhCL5, with high activity at physiological pH was suggested by Norbury et al.^28^ to function in the vitelline tissues. In *S. mansoni* and *S. japonicum*, KT inhibitors were observed between the eggshell and developing miracidia, corresponding to the vitelline mass^25,26^.

Our biochemical investigations of *F. hepatica* KT inhibitors focused on the most abundant and secreted members, FhKT1.1, FhKT1.2 and FhKT1.3. While the RSL of FhKT1.1 and FhKT1.2 contained identical sequences, the FhKT1.3 differed in having an Arg at the P1 position rather than a Leu. Consistent with our earlier reports, FhKT1.1 (and by extension FhKT1.2) exclusively inhibited cysteine proteases; these included the cathepsin L proteases from *F. hepatica,* FhCL1, FhCL2 and FhCL3, as well as host-derived mammalian cysteine proteases cathepsin L and cathepsin K (cathepsin Bs were not inhibited). FhKT1.3 also inhibited these cysteine proteases but also exhibited potent inhibitory activity against the serine protease trypsin. By substituting the P1 Leu of FhKT1.1 to an Arg (rFhKT1Leu^15^/Arg^15^) we demonstrated that this variant displayed a similar inhibitory profile to FhKT1.3 and thereby established the importance of P1 Arg for trypsin inhibition. FhKT1.3 serine protease inhibition is not broad-spectrum, however, as it did not inhibit chymotrypsin, kallikrein, and thrombin. The secreted FhKT1.3 could function alongside the intracellular FhKT4 to provide parasite defence against proteolytic attack within the duodenum, similar to trypsin-inhibiting KT proteins from other helminth parasites^29–33^.

Bozas et al.^34^ first described a Kunitz-type inhibitor in extracts of adult *F. hepatica*, termed Fh-KTM*.* Our analysis of the mass spectrometry data reported in that paper found peptides matching all the FhKT1 proteins (FhKT1.1, 1.2 and 1.3), consistent with our studies showing that these are the most abundant KTs in the parasite. Immunolocalisation studies^34^ showing that the inhibitors are dispersed throughout the parenchyma of adult worms, with evidence of trafficking towards the gut, are also consistent with our studies. Bozas et al.^34^ reported that native Fh-KTM, isolated from soluble extracts of adult parasites reduced the activity of trypsin by 93%. Since we have shown here that *F. hepatica* expresses a family of KT inhibitors, this soluble extract likely contained a mix of more than one of these KT inhibitors. However, given that FhKT1.1 and 1.2 are not inhibitors of trypsin, the trypsin inhibition recorded by Bozas et al.^34^ must be attributed to FhKT1.3.

The specific adaptation of FhKTs to cysteine proteases suggest that these inhibitors function in the regulation of the *F. hepatica* cathepsin L cysteine proteases. *F. hepatica* cysteine proteases are expressed as inactive 37 kDa zymogens that activate by auto-catalytic removal of an inhibitory 12 kDa propeptide to become a 25 kDa active mature form^35–37^. The need for an additional regulation of these proteases suggests the importance of (a) preventing uncontrolled, or leaked, auto-activation and/or (b) strict control over the proteolytic activity of the cathepsin proteases following activation and secretion. FhKT1 could be viewed as a “threshold inhibitor” as previously described for other regulatory inhibitors^38,39^. In this scenario, the ‘threshold inhibitor’ co-localises with its cognate proteases, usually at lower concentration, so that they can prevent undesirable premature activation. However, when inhibitory potential is over-run upon bulk activation of the target protease auto-catalytic activation takes place^38,39^. The *F. hepatica* cathepsin L proteases are abundant in the gastrodermal cells of the parasite as well as amongst the cargo within extracellular vesicles (EVs), microenvironments where FhKTs are also found^11,15,16,27,40–43^. Regulation of cathepsin L cysteine proteases activity ensures the majority of cathepsin L remains in an inactive form until activation is necessary, for example, upon secretion into the parasite gut lumen or release of EVs into host tissue and cells. Indeed, studies by Muiño et al.^44^ showed that a Kunitz type inhibitor co-purifies with a mature cathepsin L secreted by adult worms in culture, implying that protein–protein interactions occur between the native forms of the molecules.

Secreted FhKTs taken up into host cells, from soluble secretions or within EVs, may act as immunomodulatory proteins by targeting lysosomal cathepsin cysteine proteases. In this study, recombinant FhKT1 proteins inhibited human cathepsin L and cathepsin K cysteine proteases at sub-nanomolar concentrations indicating high potency against these enzymes. Lysosomal cathepsin L plays a critical role in MHC class II antigen processing before peptides are presented on the cell surface^45–47^. Inhibition of antigen processing and presentation impairs T cell stimulation and differentiation, resulting in diminished adaptive immunity^48,49^. Indeed, *F. hepatica* infection in mice has been shown to have a suppressive impact on the immune response^50^. Secreted and EV-contained FhKT1 proteins could be internalized by host immune cells and potentially interfere with cathepsin L-mediated antigen processing within the lysosomal compartment of the cell.

Cathepsin K has previously been shown to function in TLR-9 mediated activation of dendritic cells (DCs)^51^. Inhibition of cathepsin K in these cells results in reduced IL-6 and IL-23 production, thus preventing the induction of Th-17 cells^51,52^. Suppression of a Th-17 response was also previously observed in *F. hepatica* infection in mice^50^. Interestingly, Falcón et al.^53^ found that a < 10 kDa fraction of adult *F. hepatica* somatic extract contained a FhKT1 that suppressed LPS-activated DCs in vitro and suppressed Th1 / Th-17 allogenic response in mice. Thus, FhKT may play a role in impairing host early innate immune responses by blocking cathepsin K activity, particularly given that this protease exhibits low-level expression in DCs^51^.

Our previous studies on the structural interaction of FhKTs with cysteine proteases predicted that the P1 Leu^15^ residue of the inhibitor sits in the S2 subsite of the active site pocket^10^. In this study, we revised the model of interaction in light of our new small inhibitor binding data that showed the potential simultaneous binding of FhKT1.1 and Z-Phe-Ala-CHN_2_/E-64 in the active site of the protease. In our new docking model, FhKT1.1 binds to the S2, S1′ and S2′ pockets of FhCL1 in a somewhat similar manner to the competitive binding of cystatins to the cathepsin B of humans (PDB:3K9M). We found that Leu^15^ sits at the water-exposed interface of the S1 and S1′ subsites (near Asn^268^ of S1 and Val^245^ of S1′, Fig. 5A,B) and that replacement of the Leu^15^ with Arg^15^ in FhKT1.3 (and the variant FhKT1Leu^15^/Arg^15^) does not change binding to the cysteine proteases.

To better understand the mechanism of cysteine protease inhibition we examined the importance of Arg^19^, which is conserved in FhKT1 and FhKT1.3 and predicted to make interactions with Asp^125^ and Trp^291^ at the rim of the cysteine protease active site. While the variant rFhKT1Arg^19^/Ala^19^ still inhibited cathepsin L-like cysteine proteases, the inhibition constant (Ki) values revealed that binding was much reduced (ranging between 5- and 18-fold less potent) compared to rFhKT1 and rFhKT1.3. By contrast, and unexpectedly, we found that the variant rFhKT1Arg^19^/Ala^19^ exhibited enhanced inhibitory activity against HsCL compared to the wildtype enzyme, which we could not explain using our predicted model of interaction. Nevertheless, the data suggests that the residue Arg^19^ plays a significant and important role in the binding of the RSL.

The above data together with structural modelling of FhKT1 indicates that the RSL does not interact directly with the S1 reactive site of the cysteine protease but instead forms a bridge that sits across the S1 active site pocket blocking substrate access to the reactive Cys^132^. Pull-down experiments demonstrated that human cystatin C, a cysteine protease inhibitor of 13.3 kDa, occupies the cysteine protease active site groove and prevents binding of rFhKT1 to FhCL1 in a concentration-dependant manner. By contrast, low molecular weight cysteine protease inhibitors E-64 (360 Da) or Z-Phe-Ala-CHN_2_ (394 Da) that occupy the S1 active pocket and penetrate the S2 space^19,20^ did not prevent the binding of rFhKT1 to the enzyme active site groove. These observations prove that FhKT1 binds to the active site groove but does not penetrate deeply into the S1 or S2 sub-sites of the active site region, which is supported by our structural modelling.

In summary, phylogenetic analysis revealed a wide diversity of inhibitors amongst digenean trematode parasites that could be categorised into seven distinct groups. *F. hepatica* expresses a temporally-regulated family of Kunitz-like inhibitors with unique cysteine protease-inhibiting activity. *F. hepatica* also expresses several broad-spectrum cystatins^54^ that inhibit the parasite cathepsin L-like cysteine proteases indicating the importance for the parasite to tightly control the activity of these enzymes during migration, growth and development. Moreover, inhibition of key host lysosomal cathepsin L-like cysteine proteases involved in antigen processing which may be a means of controlling host responses to parasite molecules. These putative pivotal roles in host-parasite interaction position the FhKT1 inhibitors as viable vaccine and drug targets against the globally important zoonotic parasite *F. hepatica*.

## Experimental procedures

### Parasite material and excystment protocols

*F. hepatica* metacercariae (Italian isolate; Ridgeway Research, UK) were excysted and cultured in RPMI 1640 medium containing 2 mM l-glutamine, 30 mM HEPES, 0.1% (w/v) glucose, and 2.5 µg/ml gentamycin for up to 48 h as described by Cwiklinski et al.^4^. Adult *F. hepatica* parasites were recovered from livers of naturally infected sheep at a local abattoir, washed with PBS (containing 0.1% glucose) and cultured in the same medium for 5 h. The parasite culture media (parasite ES proteins) were collected, centrifuged at 300×*g* for 10 min and at 700×*g* for 30 min and stored at − 80 °C. The adult parasite somatic extract was isolated by homogenizing parasites in 500 μl PBS and centrifugation at 4500×*g* for 10 min, and the supernatant stored at − 80 °C.

### Identification of a Kunitz-type protease inhibitor gene family and phylogenetic analysis

The identification of the *F. hepatica* Kunitz-type (KT) inhibitor gene (*fhkt*) family was performed using BLAST analysis against the *F. hepatica* genome^3,55^ using previously identified *F. hepatica* Kunitz sequences (FhKT1^10^; Fh_Contig2704^56^,) followed by manual assessment to identify the characteristic and conserved six cysteine residues that form three distinctive disulphide bridges. In addition, the *F. hepatica* gene models^3^ putatively annotated using in silico tools (Uniprot, Gene Ontology (GO), and InterProScan) were screened for ‘kunitz-type protein’ within their descriptive annotations. Homologous trematode KT sequences were retrieved using BLAST, manual curation and putative annotation as above from publically available transcriptome and genome databases, from the following databases: (a) WormBase ParaSite^55,57^): *Clonorchis sinensis* (PRJDA72781), *Opisthorchis viverrini* (PRJNA222628), *Echinostoma caproni* (PRJEB1207), *Schistosoma haematobium* (PRJNA78265), *Schistosoma japonicum* (PRJEA34885) and *Schistosoma mansoni* (PRJEA36577); (b), Trematode.net^58,59^: *Paragonimus westermani* (PRJNA219632); (c) the adult *Fasciola gigantica* transcriptome^60,61^. Maximum likelihood trees were constructed with the trematode-specific KT sequences (Supplementary Table S1) using MEGA v4.0 with the nucleotide sequence corresponding to central structural domain of the KTs (first to last conserved Cys residue, see Fig. 1), with bootstrap values calculated from 1000 iterations.

### Transcriptomic and proteomic expression analysis of the *F. hepatica* Kunitz gene family

Differential gene transcription of the *F. hepatica* KT genes was investigated using the available *F. hepatica* transcriptome data (European Nucleotide Archive accession number PRJEB6904) as described by^3^, represented as the log of the number of transcripts per million (log TPM; Fig. 2) and fold change relative to the metacercariae stage (Supplementary Fig. S3).

Quantitative real time PCR (qPCR) analysis of the *fhkt1* genes was carried out on NEJs cultured for 0 h, 6 h, 10 h, 24 h and 48 h in RPMI 1640 medium containing 2 mM l-glutamine, 30 mM HEPES, 0.1% glucose, 2.5 µg/ml gentamycin and 10% foetal calf serum (ThermoFisher Scientific). Total RNA extraction and cDNA synthesis was carried out as per Cwiklinski et al.^4^. Primers were designed to amplify all three genes based on the genomic sequence data. qPCR reactions were performed in 20 µl reaction volumes in triplicate, using 1 µl cDNA diluted 1:2, 10 µl of Platinum SYBR Green qPCR SuperMix-UDG kit (ThermoFisher Scientific) and 1 µM of each primer (*fhkt1*Forward (5′-ATCCAAAAACGATGTCTTCTTCCGG-3′) and *fhkt1*Reverse (5′-TTGGAATCGAAAACCACAGTT-3′). A negative control (no template) was included in each assay. qPCR was performed using a Rotor-Gene thermocycler (Qiagen), with the following cycling conditions: 95 °C: 10 min; 40 cycles: 95 °C:10 s, 54 °C:15 s, 72 °C: 20 s; 72 °C: 5 min. Relative expression analysis was performed manually using Pfaffl's Augmented ΔΔCt method^62^ whereby the comparative cycle threshold (Ct) values of samples of interest were compared normalised to the housekeeping gene, Glyceraldehyde 3-phosphate dehydrogenase (GAPDH). In order for this method to be valid, amplification efficiencies of individual reactions were verified using the comparative quantification package within the Rotor-Gene Q software v2.1.0. Annealing temperatures and melt-curve analysis was also carried out to check for single DNA products produced by these primer sets. Results were analysed using One Way ANOVA (P-value < 0.05 was deemed statistically significant) and visualised using version 6.00 for Windows, GraphPad Software (https://www.graphpad.com/scientific-software/prism/).

*F. hepatica* proteomic datasets were interrogated for FhKT1 proteins within the secreted products (ES proteins) from NEJs (ProteomeXchange Consortium repository: PXD007255;^4^), adult parasites (ProteomeXchange Consortium repository:PXD002570;^11^) and the extracellular vesicles (EVs) isolated from adult *F. hepatica* ES (ProteomeXchange Consortium repository:PXD002570;^11^). The number of unique peptides identified was validated using Scaffold (version 4.3.2)^11^.

### Analysis of the *fhkt1.3* cDNA

Total RNA was extracted from a single adult fluke using miRNeasy Mini Kit (Qiagen) and cDNA synthesised using High capacity cDNA reverse transcription kit (ThermoFisher Scientific). Primers were designed to bind to a consensus nucleotide sequence encoding the signal peptides identified by SignalP v4.1, in *fhkt1.1* and *fhkt1.2* (fhkt1SP: 5′ATGCGTTGTTTCACAATCGCC 3′) and to the nucleotide sequence corresponding to the conserved residues at the start of the conserved KT domain (fhkt1F: 5′AACGATGTCTTCTTCCGGTCG 3′). The reverse primer corresponded to the conserved 3′ end of the *fhkt1* nucleotide sequence (fhkt1R: 5′ TTATTGGAATCGAAAACCACAGTTG 3′). PCR products were gel purified (QIAquick Gel Extraction Kit, Qiagen) and transformed into TOP10 (BL21) *Escherichia coli* competent cells (TOPO cloning system, ThermoFisher Scientific). Plasmid DNA from 20 transformants were sequenced by Source Bioscience (UK).

### Whole-mount NEJ Immunolocalization of FhKT1 by confocal microscopy

NEJs were fixed with 4% paraformaldehyde in 0.1 M PBS and then incubated in 100 mM PBS containing anti-recombinant FhKT1 antiserum at a 1:500 dilution, overnight at 4 °C, followed by three washes in AbD^4,10^. NEJ were then incubated in a 1:200 dilution of the secondary antibody, fluorescein isothiocyanate (FITC)-labelled goat anti-rabbit IgG (Sigma-Aldrich) overnight at 4 °C. To counter-stain muscle tissues, NEJs were incubated in AbD containing 200 μg/ml phalloidintetramethylrhodamine isothiocyanate (TRITC) overnight at 4 °C. NEJs were whole-mounted in a 9:1 glycerol solution containing 100 mM propyl gallate and viewed using confocal scanning laser microscopy (Leica TCS SP5; Leica Microsystems, UK) under the HCX PL APO CS × 100 oil objective lens.

### Immunolocalization of FhKT1 in adult *F. hepatica* by fluorescence light microscopy

Adult flukes were fixed in paraformaldehyde at 4 °C overnight, washed with PBS, dehydrated with ethanol and embedded in JB-4 resin (Sigma-Aldrich)^4,10^. Sections (2 μm) were incubated in either an anti-peptide antibody raised in mice to the following FhKT1.1 amino acid sequence, Cys-Glu-Gly-Asn-Asp-Asn-Arg-Phe-Asp-Ser-Lys-Ser-Ser-Cys, or pre-immune sera, each at a 1:500 dilution. Sections were then washed three times in PBS with 0.5% Triton X-100 for 30 min and incubated in a 1:500 dilution of the secondary antibody, fluorescein isothiocyanate (FITC)-labelled goat anti-mouse immunoglobulin (Sigma-Aldrich). After three washes in PBS with 0.5% Triton X-100 for 30 min sections were dried and coverslips mounted using glycerol:PBS (9:1) containing 100 mM propyl gallate. Sections were viewed using a Leica DM 2500 light microscope under the HCX PL FLUOSTAR × 10 and × 40 lenses.

### Molecular modelling

The homology model of FhKT1.1 (built based on the BPTI crystal structure) was taken from Smith et al.^10^ to conduct docking studies using the protein–protein docking server, ZDOCK^63^. Given that FhKT1 does not block the binding of the competitive antagonist, Z-Phe-Ala-CHN2 to FhCL1, the initial binding hypothesis of FhKT1-FhCL1 that we proposed in Smith et al.^10^ was re-examined. ZDOCK produced ten docking solutions involving binding to S1 and S2 pockets as well as S1′ and S2′ pockets. A complex of FhCL1 bound to Z-Phe-Ala-CHN_2_ was generated by superimposing the structure of FhCL1 with the crystal structures of human cathepsins L and K bound to diazomethylketone or E-64 inhibitors at S1 and S2 pockets, respectively (Pdb codes: 3OF9 and 1ATK) using Maestro 10.2 (Schrödinger^64^). The ZDOCK structure of FhKT1.1 that does not overlap with the S1, S2 and S3 pockets of FhCL1 was selected as a starting conformation for energy optimization. The FhCL1 complex bound to FhKT1 and Z-Phe-Ala-CHN_2_ was subjected to an optimization procedure involving 2000 step minimization and 200 ps dynamic simulations using the MacroModel module of Schrodinger software^64^. Graphical representations were created using Schrödinger software (LLC. Maestro, Version 2018–4; https://www.schrodinger.com/).

### Production of functional recombinant Kunitz-type inhibitors in the methylotrophic yeast *Pichia pastoris*

Recombinant proteins were expressed in *P. pastoris* with a C-terminal His-tag as previously described in Smith et al.^10^. Protein yield was quantified by measuring the absorbance at A_280_ and using the Protein calculator^65^. Protein purity was visualised by NuPAGE Novex 4–12% BisTris protein gel (ThermoFisher Scientific).

### Determination of FhKT1.1, FhKT1.3 and FhKT1Arg^19^/Ala^19^ protease inhibition profile and kinetics

Enzymes included bovine trypsin, bovine chymotrypsin, human cathepsin B, human cathepsin L, and human cathepsin S (all Sigma-Aldrich) and human cathepsin K (Enzo Life Sciences). Purified *F. hepatica* cathepsin L1 (FhCL1), *F. hepatica* cathepsin L2 (FhCL2) and *F. hepatica* cathepsin L3 (FhCL3)^66^ and recombinant FhKT1 and FhKT1Leu^15^/Arg^15^^10^ were expressed as active recombinant proteins in *P. pastoris*. Reaction conditions and substrates employed for measuring the activity of each protease were as reported by Smith et al.^10^. Additionally, rFhCL3 activity was measured using the fluorogenic substrate Z-Gly-Pro-Arg-NHMec (20 μM).

KT inhibitors (2 μM) was incubated with each protease in a 100 μl volume of reaction buffer for 15 min at 37 °C. Reaction were brought to 200 μl with the addition of fluorogenic substrate dissolved in reaction buffer and proteolytic activity measured as RFU (relative fluorescent units) using a PolarStar Omega spectrophotometer (BMG LabTech, UK). Inhibition constants were determined using the Morrison equation for tight-binding inhibition as previously described^10^.

### rFhKT1 and rFhKT1.3 inhibition of *F. hepatica* cysteine proteases in somatic extract and ES proteins

Decreasing concentrations of rFhKT1 and rFhKT1.3 were incubated for 10 min at 37 °C with 2 μl of *F. hepatica* somatic extract or ES diluted in 100 mM sodium acetate buffer pH5.5, containing 2 mM DTT and 0.01% Brij L23. To measure any remaining cysteine protease activity in the presence of the inhibitors, 100 μl of reaction buffer (sodium acetate buffer pH5.5, containing 2 mM DTT and 0.01% Brij L23) containing Z-Phe-Arg-NHMec (20 μM) was added and monitored using a PolarStar Omega spectrophotometer. As a positive control, assays used decreasing concentrations of the cysteine protease inhibitor E-64.

### Inhibition competition and pulldown assays of FhKT1 binding to native cathepsin L-like cysteine proteases

To examine the ability of cysteine protease inhibitors to compete for active site binding with FhKT1, competition assays were carried out using three different cysteine proteases inhibitors, E-64, Z-Phe-Ala-CHN_2_ and recombinant human cystatin C (Sigma-Aldrich). The cysteine protease inhibitors were added to adult *F. hepatica* ES proteins (~ 20 μg protein) at the following final concentrations; E-64 and Z-Phe-Ala-CHN_2_: 1 mM, 100 μM, 5 μM, 1 μM, 100 nM, 10 nM and 1 nM; recombinant human cystatin C: 500 nM, 250 nM, 100 nM, 50 nM, 10 nM and 1 nM. After 15 min at 37 °C, two μl of each combined *F. hepatica* ES proteins and inhibitor sample was added to 98 μl of sodium acetate buffer (containing 2 mM DTT and 0.01% Brij L23), then brought to 200 μl upon the addition of the fluorogenic peptide substrate Z-Phe-Arg-NHMec (20 μM) dissolved in the sodium acetate buffer. Fluorogenic assays were carried out in triplicate using a PolarStar Omega spectrophotometer, reported at RFU.

The remainder of each *F. hepatica* ES proteins/inhibitor mix was added to 1 μM of rFhKT1.1 and incubated at 37 °C for 30 min. Following the addition of 10 μl of Ni–NTA beads, the samples were incubated at room temperature with rotation for 30 min. The Ni–NTA beads were then pelleted by centrifugation at 100×*g* in a bench top microcentrifuge, washed twice with wash buffer and the bound proteins eluted in 20 μl of elution buffer. Eluted proteins were analysed on NuPAGE Novex 4–12% BisTris gels (ThermoFisher Scientific), stained with Biosafe Coomassie (BioRad), and imaged using a G:BOX Chemi XRQ imager (Syngene). Additional inhibition competition assays were performed with rFhKT1Arg^19^/Ala^19^ and Z-Phe-Ala-CHN_2_-inhibited cathepsin L ES proteases as described above for rFhKT1.

## Supplementary information

Supplementary Information

## Data Availability

The transcriptome data sets supporting the conclusions of this article are available in the European Nucleotide Archive repository, PRJEB6904; http://www.ebi.ac.uk/ena/data/view/PRJEB6904, previously reported by Cwiklinski et al*.*^3^. The mass spectrometry proteomics data analysed as part of this study have been deposited to the ProteomeXchange Consortium via the PRIDE partner repository with the following data set identifiers (a) NEJ specific datasets^4^: PXD007255 and 10.6019/PXD007255; (b) adult ES and EV datasets^11^: PXD002570 and 10.6019/ PXD002570.
