# Supplementary material for: An atypical and functionally diverse family of Kunitz-type cysteine/serine proteinase inhibitors secreted by the helminth parasite Fasciola hepatica
Source: Sci Rep. 2020 Nov 26;10:20657. doi: 10.1038/s41598-020-77687-7 (PMC7692546; doi:10.1038/s41598-020-77687-7)

An atypical and functionally diverse family of Kunitz-type cysteine/serine proteinase inhibitors secreted by the helminth parasite *Fasciola hepatica*

David Smith[1,2], Krystyna Cwiklinski[1,3], Heather Jewhurst[1,3], Irina G. Tikhonova[4], John P. Dalton[1,3]

Author affiliations:

1. School of Biological Sciences, Queen's University Belfast BT9 7BL, Belfast, Northern Ireland, United Kingdom; 2. Present address: Moredun Research Institute, Pentland Science Park, Penicuik, Midlothian, Scotland, United Kingdom; 3. Centre of One Health and Ryan Institute, School of Natural Sciences, National University of Ireland Galway, Galway, Ireland; 4. School of Pharmacy, Medical Biology Centre, Queen's University Belfast, Belfast BT9 7BL, Northern Ireland, United Kingdom.

Supplementary Information

Supplementary Table S1. Trematode Kunitz type inhibitor gene sequences used for phylogenetic analysis.

Supplementary Table S2. FhKT proteins present in the excretory/secretory products of NEJ and adult *F. hepatica* represented as Normalized Spectral Abundance Factor (NSAF).

Supplementary Figure S1. Schematic representation of the *F. hepatica* Kunitz-type gene structure

Supplementary Figure S2. Phylogenetic analysis of helminth Kunitz-type inhibitors.

Supplementary Figure S3. Graphical representation of differential gene expression represented as fold change compared to the metacercariae stage.

Supplementary Figure S4. Immunolocalization of FhKT1 proteins in the reproductive organs of adult *F. hepatica.*

Supplementary Figure S5. Immunolocalization of FhKT1 proteins in the gut of adult *F. hepatica.*

Supplementary Figure S6. rFhKT1.1Arg^{19}/Ala^{19} binding to the active site groove of native cathepsin Ls is not prevented by Z-Phe-Ala-CHN_2.

Supplementary Figure S7. Full length gel photos as shown in (A) Fig. 6; (B) Fig. 9B, D and F; (C) Supplemental Figure 5.

Supplementary Table S1: Trematode Kunitz type inhibitor gene sequences used for phylogenetic analysis

Nomenclature on Phylogram	Species	Gene/Scaffold ID	P1-P4'
**Group A**			
fgkt1	*Fasciola gigantica*	Contig12	LGGIR
fhkt1.2	*Fasciola hepatica*	BN1106_s318B000274	LGGIR
fhkt1.1	*Fasciola hepatica*	BN1106_s8826B000029	LGGIR
fhkt1.3	*Fasciola hepatica*	BN1106_s11518B000016	RGGIR
eckt1	*Echinostoma caproni*	ECPE_0001647201	FRGGI
eckt2	*Echinostoma caproni*	ECPE_0001016301	LAIHY
eckt3	*Echinostoma caproni*	ECPE_0001278301	LAIHY
fgkt2	*Fasciola gigantica*	Contig38896	LAIRP
fhkt2	*Fasciola hepatica*	BN1106_s6608B000014	LAIRP
**Group B**			
cskt1	*Clonorchis sinensis*	csin108828	HENYT
fhkt3	*Fasciola hepatica*	scaffold5597	SEHIT
cskt2	*Clonorchis sinensis*	csin107698	AENLR
eckt4	*Echinostoma caproni*	ECPE_0001105001	RGYHV
eckt5	*Echinostoma caproni*	ECPE_0000795001	RAAIT
sjkt1	*Schistosoma japonicum*	Sjp_0020270	RASLL
shkt1	*Schistosoma haematobium*	MS3_09801	RSKLH
smkt3	*Schistosoma mansoni*	Smp_139840	RASFN
**Group C**			
sjkt4	*Schistosoma japonicum*	Sjp_0097640	RYNYH
sjkt5	*Schistosoma japonicum*	Sjp_0117580	GNNST
sjkt6	*Schistosoma japonicum*	Sjp_0024620	LKRHP
shkt3	*Schistosoma haematobium*	MS3_09688	LQNIP
sjkt7	*Schistosoma japonicum*	Sjp_0024630	LHNKP
shkt4	*Schistosoma haematobium*	MS3_10748	LQKKP
smkt2	*Schistosoma mansoni*	Smp_179120	LQNKP
**Group D**			
sjkt2	*Schistosoma japonicum*	Sjp_0030350	RASIQ
cskt4	*Clonorchis sinensis*	csin103940	RGDVT
ovkt2	*Opisthorchis viverrini*	T265_11148	FVTAT
**Group E**			
cskt6	*Clonorchis sinensis*	csin112642	KAYMP
cskt7	*Clonorchis sinensis*	csin106214	LASMP
ovkt1	*Opisthorchis viverrini*	T265_11147	RAMIP
cskt3	*Clonorchis sinensis*	csin102310	RAMIP
eckt8	*Echinostoma caproni*	ECPE_0000615701	FHIFI
fhkt4	*Fasciola hepatica*	BN1106_s3911B000104	RGSFP
eckt6	*Echinostoma caproni*	ECPE_0001134901	GANIL

fhkt5	*Fasciola hepatica*	BN1106_s4272B000063	LAHIP
fgkt5	*Fasciola gigantica*	Contig26461	LALVP
eckt7	*Echinostoma caproni*	ECPE_0001663401	HTLLL
**Group F**			
pwkt5	*Paragonimus westermani*	comp19852	SDSIT
pwkt4	*Paragonimus westermani*	comp14876	GESLT
pwkt3	*Paragonimus westermani*	comp20534	MGHST
pwkt1	*Paragonimus westermani*	comp21326	RALIK
pwkt2	*Paragonimus westermani*	comp15715	RALMK
**Group G**			
smkt1	*Schistosoma mansoni*	Smp_147730	RALLK
shkt2	*Schistosoma haematobium*	MS3_09690	RALIK
cskt5	*Clonorchis sinensis*	csin102323	NFRTR
sjkt3	*Schistosoma japonicum*	Sjp_0076670	RGYFR
eckt9	*Echinostoma caproni*	ECPE_0000445201	SQFIT

Supplementary Table S2: FhKT proteins present in the excretory/secretory products of NEJ and adult *F. hepatica* represented as Normalized Spectral Abundance Factor (NSAF).

Inhibitor	NEJ 1h**	NEJ 3h**	NEJ 24h**	Adult	Adult EVs
FhKT1.1	-	-	0.00330	-	0.00416
FhKT1.2	0.01131	0.00482	0.00967	0.18816	0.01258
FhKT1.3	0.00447	0.00293	-	0.00615	-

**Average NSAF value from triplicate samples

Supplementary Figure S1. Schematic representation of the *F. hepatica* Kunitz-type gene structure. Exons are represented by the coloured boxes and the introns are depicted as a black line. The nucleotide sequence encoding the signal peptide (exon 1) and Kunitz domain (exon 2) is represented by the blue exon and red exons, respectively. *fhkt1.3* consists of only one exon, with no sequence encoding a signal peptide. Scale bar indicates the length of 100 nucleotide base pairs. The size of the respective introns in the *F. hepatica* genome assembly (PRJEB6687) is shown.

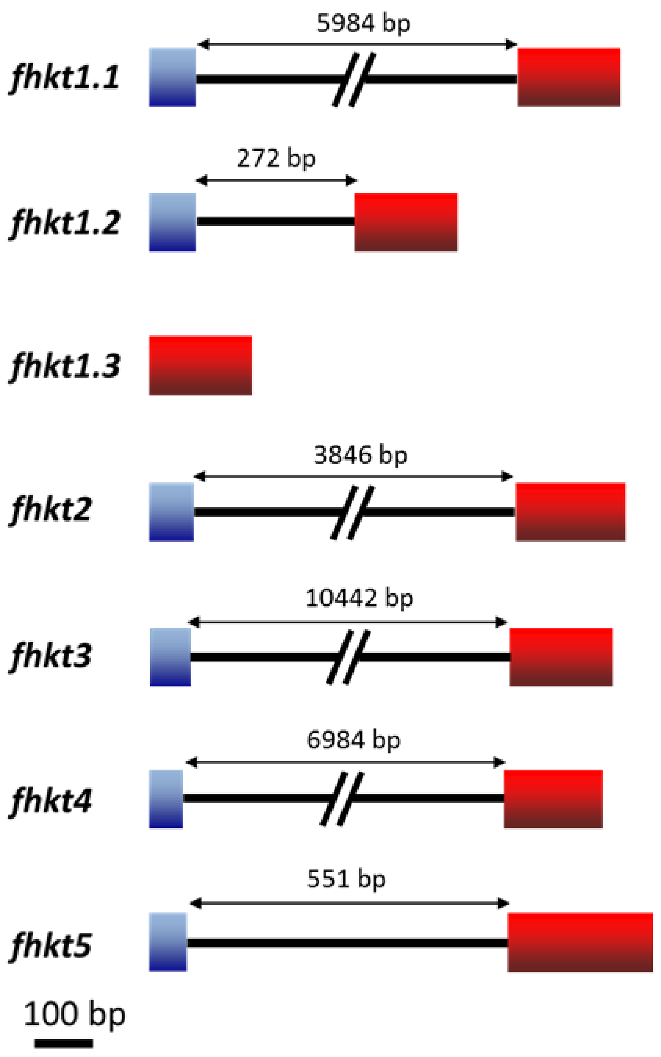

Supplementary Figure S2. Phylogenetic analysis of helminth Kunitz-type inhibitors. Maximum-likelihood phylogenetic tree computed using 1000 bootstrap replicates based on the sequence encoding the kunitz domain between cysteine residues 1 and 6 (Cys^1 and Cys^6), from nine helminth species: *Clonorchis sinensis* (*cskt1-7*), *Echinostoma caproni* (*eckt1-9*), *Fasciola hepatica* (*fhkt1-5*), *F. gigantica* (*fgkt1, 2* and *5*), *Opisthorchis viverrini* (*ovkt1-2*), *Paragonimus westermani* (*pwkt1-5*), *Schistosoma haematobium* (*shkt1-4*), *S. japonicum* (*sjkt1-7*) and *S. mansoni* (*smkt1-3*). Bootstrap values >50% are shown. The green coloured blocks highlight the presence of the *F. hepatica* KT sequences within 3 distinct groups formed by phylogenetic analysis (A, B, and E). The black lines represent the other clusters generated by phylogenetic analysis that do not contain *F. hepatica* sequences (clusters C, D, F and G). The accession numbers of the sequences represented by this phylogenetic tree are included in Supplementary Table S1.

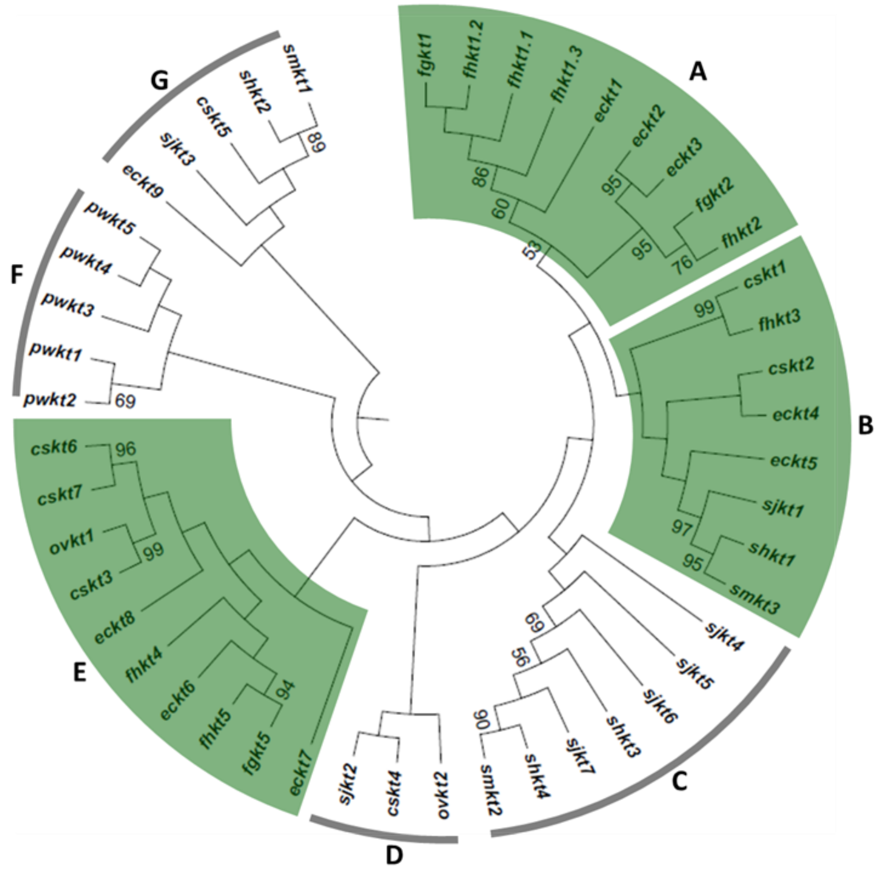

Supplementary Figure S3. Graphical representation of differential gene expression of the FhKT family represented as log fold change compared to the metacercariae life cycle stage. Differential expression was calculated as reported by Cwiklinski et al. [3] using negative binomial model of successive developmental stages relative to metacercariae and tagwise dispersion estimated from all samples in edge R. (*p*<0.01: **; *p*<0.001: ***).

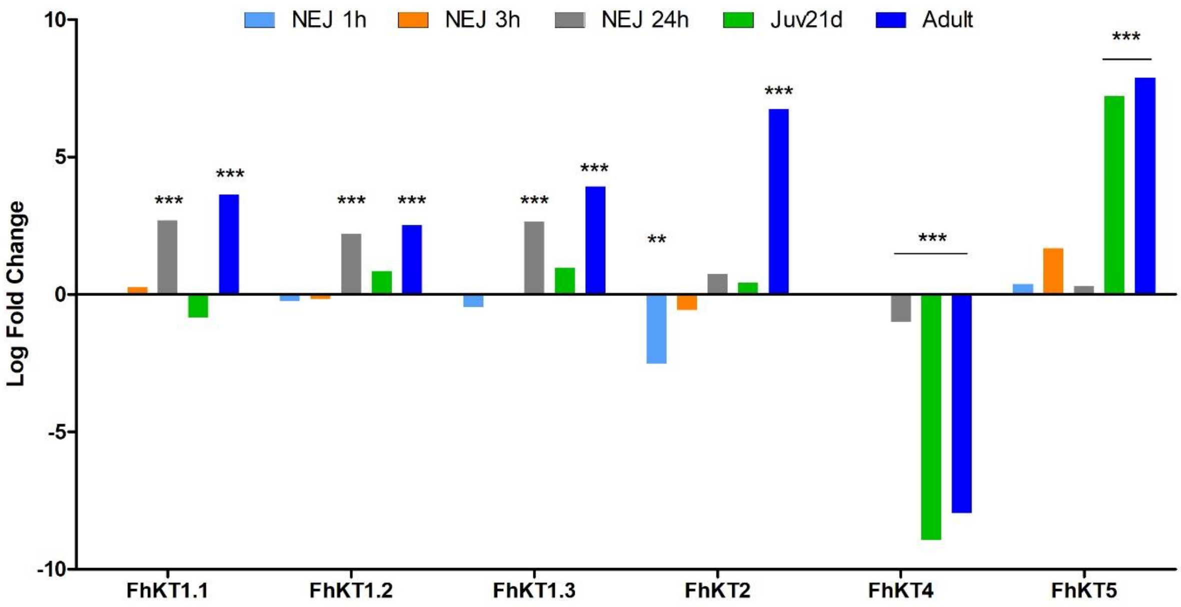

Supplementary Figure S4. Immunolocalization of FhKT1 proteins in the reproductive organs of adult *F. hepatica*. Serial sections through a JB-4 embedded adult *F. hepatica* were stained with Toluidine Blue or anti-FhKT1 antibodies. Toluidine Blue staining highlights the vitelline glands (A) and ovaries containing parasite eggs (E) (10x). Sections probed with mouse pre-immune serum (negative control) and stained with anti-mouse FITC show light background fluorescence in the testes (B) and eggshell (F) (10x). Serial sections probed with polyclonal anti-FhKT1 antibodies show strong staining in the vesicular structures within the vitelline cells contained in the vitelline glands (C) (10x), as also shown at higher magnification of 40x (D). Intense staining was also observed in the vitelline cell-derived yolk mass within eggs (G and H, 10X and 100x, respectively). E: egg; G: gut; T: testes; Te: tegument; V: vitelline glands containing vitelline cells. Scale bar: panels A-C and E-G, 200 μm; panels D and H, 50 μm.

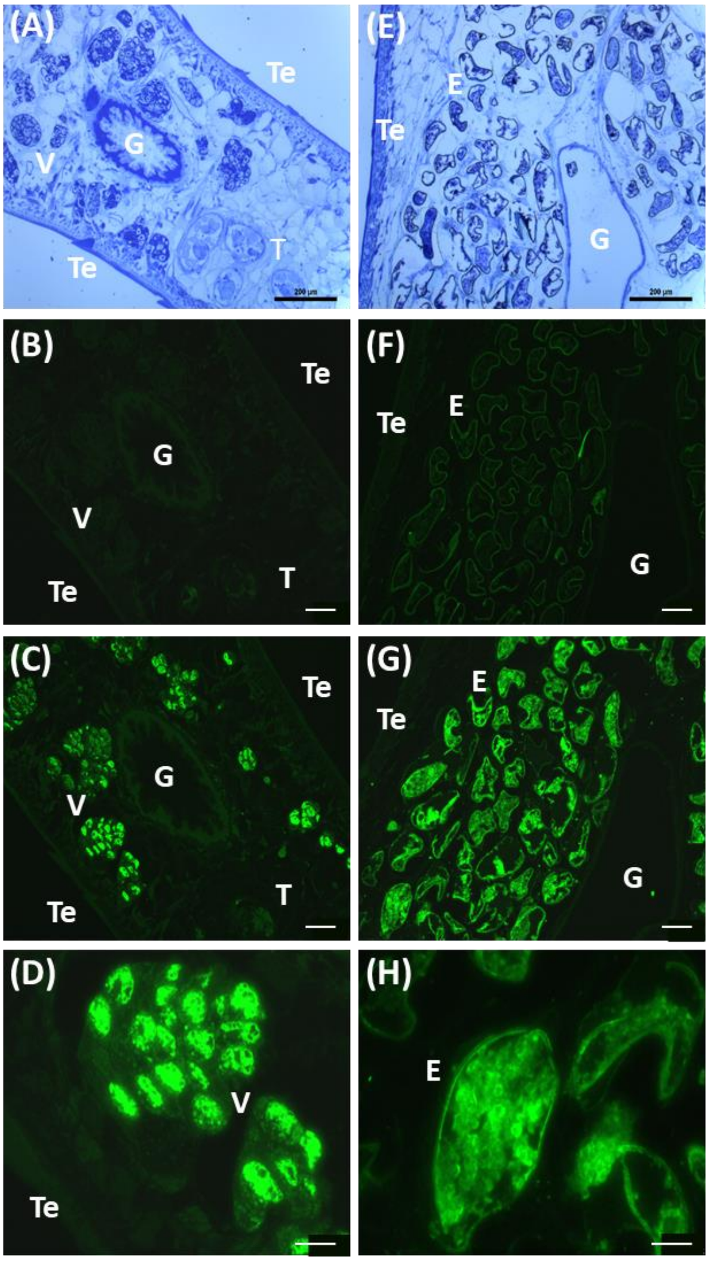

Supplementary Figure S5. Immunolocalization of FhKT1 proteins in the gut of adult *F. hepatica.* (A) Cross-section of a JB-4 section of adult *F. hepatica* stained with Toluidine Blue highlighting the digestive gut, parenchyma, tegument and the ventral sucker. (B) Section probed with mouse pre-immune serum (negative control) (C-D) or anti-FhKT1 antibodies followed by with anti-mouse-FITC. Visible staining in the within the ventral sucker and parenchymal cell bodies that are concentrated around near the gut, with diffuse staining throughout the parenchyma and gut (C and D, 10x and 40 x, respectively). G, gut; PC, Parenchymal cell body; Te, tegument; VS, ventral sucker. Scale bar: panels A-C, 200 µm; panel D, 50 µm.

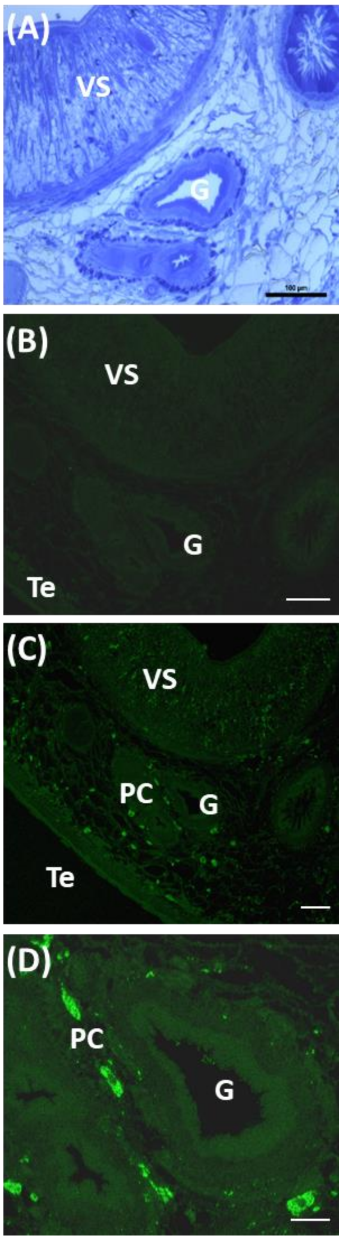

Supplementary Figure S6. rFhKT1.1Arg^{19}/Ala^{19} binding to the active site groove of native cathepsin Ls is not prevented by Z-Phe-Ala-CHN_2 (A) Cysteine protease activity within adult *F. hepatica* excretory/secretory (ES) products measured in the presence of Z-Phe-Ala-CHN_2, at a concentration ranging from 1 nM to 100µM (% Activity, relative to the cysteine protease activity of ES containing no inhibitor ± SD). (B) rFhKT1.1Arg^{19}/Ala^{19} (10 µM) was added to replicate reaction samples and then pull-down using NTA-beads. rFhKT1.1Arg^{19}/Ala^{19} (black arrow) is not prevented from interacting with the cathepsin L cysteine proteases by Z-Phe-Ala-CHN_2 (white arrows).

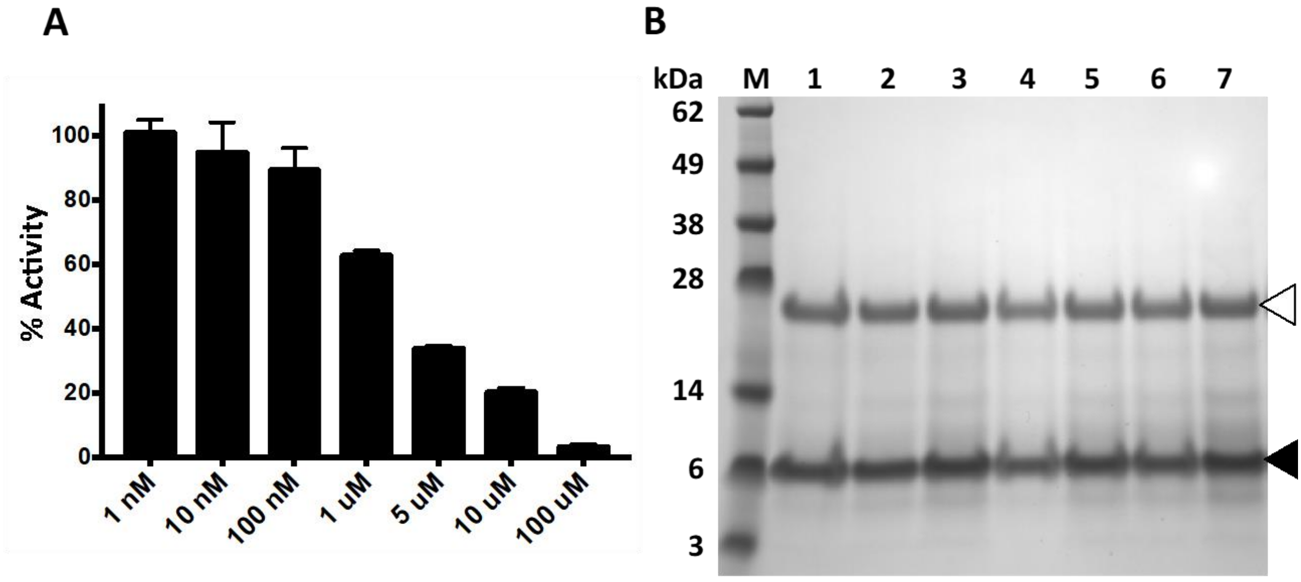

Supplementary Figure S7. Full length gel photos as shown in (A) Fig. 4A; (B) Fig. 7B, D and F; (C) Supplemental Figure 6B.

(A) Fig 4A

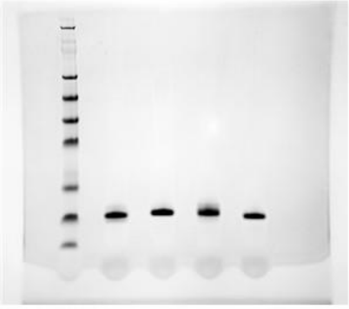

(B) Fig. 7B

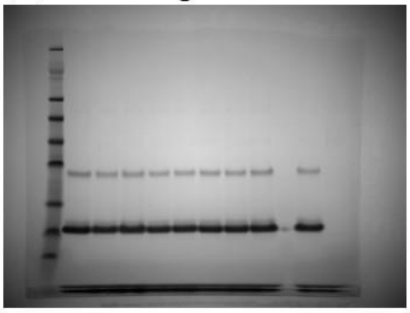

Fig. 7D

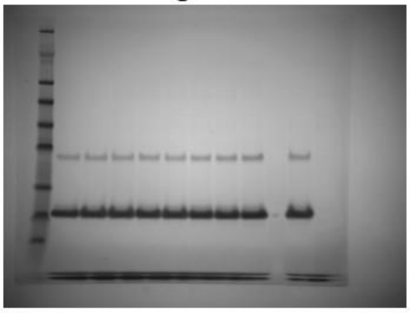

Fig. 7F

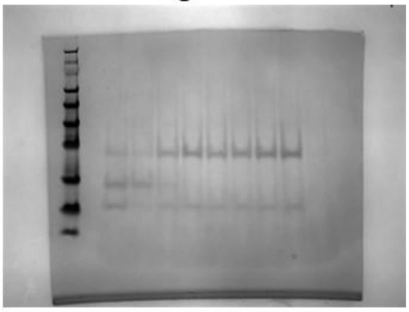

(C) Supplementary Fig. 6B

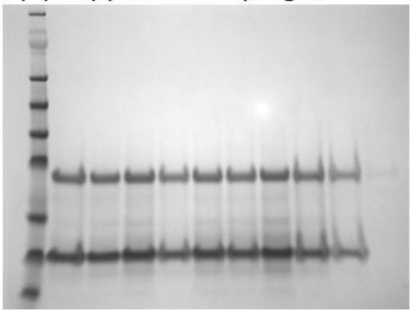

Supplement: Supplementary file 1 — Supplementary Information [file 41598_2020_77687_MOESM1_ESM.pdf]
